# Modular Prodrug‐Engineered Oxygen Nano‐Tank With Outstanding Nanoassembly Performance, High Oxygen Loading, and Closed‐Loop Tumor Hypoxia Relief

**DOI:** 10.1002/advs.202405583

**Published:** 2024-07-10

**Authors:** Fujun Yang, Shumeng Li, Qingyu Ji, Hongyuan Zhang, Mingyang Zhou, Yuequan Wang, Shenwu Zhang, Jin Sun, Zhonggui He, Cong Luo

**Affiliations:** ^1^ Department of Pharmaceutics, Wuya College of Innovation Shenyang Pharmaceutical University Shenyang 110016 P. R. China; ^2^ Joint International Research Laboratory of Intelligent Drug Delivery Systems, Ministry of Education Shenyang Pharmaceutical University Shenyang 110016 P. R. China; ^3^ Department of Pharmaceutical Analysis, School of Pharmacy Shenyang Pharmaceutical University Shenyang 110016 P. R. China; ^4^ Department of Chemistry University of Pennsylvania Philadelphia Pennsylvania 19104‐6323 USA

**Keywords:** carrier‐free nanoassembly, fluorination prodrug, hypoxia alleviation, oxygen supply/consumption modulation, photodynamic therapy

## Abstract

The clinical translation of tumor hypoxia intervention modalities still falls short of expectation, restricted by poor biocompatibility of oxygen‐carrying materials, unsatisfactory oxygen loading performance, and abnormally high cellular oxygen consumption‐caused insufficient hypoxia relief. Herein, a carrier‐free oxygen nano‐tank based on modular fluorination prodrug design and co‐assembly nanotechnology is elaborately exploited, which is facilely fabricated through the molecular nanoassembly of a fluorinated prodrug (FSSP) of pyropheophorbide a (PPa) and an oxygen consumption inhibitor (atovaquone, ATO). The nano‐tank adeptly achieves sufficient oxygen enrichment while simultaneously suppressing oxygen consumption within tumors for complete tumor hypoxia alleviation. Significant, the fluorination module in FSSP not only confers favorable co‐assemblage of FSSP and ATO, but also empowers the nanoassembly to readily carry oxygen. As expected, it displays excellent oxygen carrying capacity, favorable pharmacokinetics, on‐demand laser‐triggerable ATO release, closed‐loop tumor hypoxia relief, and significant enhancement to PPa‐mediated PDT in vitro and in vivo. This study provides a novel nanotherapeutic paradigm for tumor hypoxia intervention‐enhanced cancer therapy.

## Graphical abstract

A credible oxygen nano‐tank is elaborately exploited to potentiate oxygen‐dependent photodynamic therapy against cancer. Specifically, a novel fluorination prodrug (FSSP) of PPa is designed and synthesized. FSSP is find to readily co‐assemble with ATO (an oxygen consumption inhibitor) into a carrier‐free oxygen nano‐tank with remarkable capabilities in oxygen supply and consumption inhibition capacity.

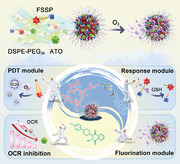

## 1 Introduction

For decades, external beam energy therapy has continually evolved for cancer treatment, including radiotherapy and photodynamic therapy (PDT).^[^ ^1^ ^]^ Photodynamic photosensitizers produce large amounts of cytotoxic singlet oxygen under laser irradiation, which usually has a stronger tumor‐killing effect than radiotherapy.^[^ ^2^ ^]^ However, owing to the weak tissue penetration of light, PDT is used mostly to treat tumors located in superficial part of human bodies or those located in gastrointestinal tract, such as breast cancer, head‐and‐neck cancer and colon cancer.^[^ ^1^, ^2^, ^3^ ^]^ These malignant solid tumors frequently suffer from hypoxia dilemma, which substantially undermine the treatment outcomes of clinically available therapeutics including PDT, chemotherapy, radiotherapy, and immunotherapy.^[^ ^4^ ^]^ Particularly, tumor hypoxia has been widely recognized one of the stubbornest obstacles hindering the clinical translation and application of oxygen‐dependent PDT modalities.^[^ ^5^, ^6^, ^7^, ^8^ ^]^ Moreover, tumor hypoxia is highly correlated with the unfavorable clinical prognosis, even leading to a heightened incidence of tumor recurrence and metastasis.^[^ ^9^, ^10^ ^]^ Over decades, a multitude of pivotal factors have been elucidated as contributors to tumor hypoxia, including aberrant tumor vasculatures, blood flow fluctuation, and limited oxygen diffusion within the confines of tumor tissues.^[^ ^11^, ^12^ ^]^ Moreover, the unrestrained proliferation characteristic of tumor cells is linked to crazy oxygen consumption, significantly exacerbating the prevailing hypoxic predicament within solid tumors.^[^ ^13^ ^]^ Consequently, oxygen concentrations within solid tumors plummet to levels below 2.5 mmHg pO_2_, a striking contrast to the average of 40 mmHg pO_2_ observed in healthy tissues.^[^ ^4^ ^]^ Emerging evidence underscores the remarkable adaptability of tumor cells in down‐regulating their oxygen utilization as a survival response to severe oxygen deprivation within hypoxic environments.^[^ ^14^, ^15^, ^16^ ^]^ Our observations also revealed a substantial reduction in the oxygen consumption rate (OCR) of 4T1 breast cancer cells and CT26 colon adenocarcinoma cells under hypoxia conditions. Significant, we further found that exogenous oxygen supplementation to hypoxic 4T1 and CT26 cells elicited a rapid surge in cellular OCR, swiftly reverting to initial hypoxia levels. These findings heralded that oxygen consumption inhibition hold equal significance to oxygen supplementation in the pursuit of effective alleviation of tumor hypoxia.

In the last decades, considerable endeavors have been devoted to realizing exogenous oxygen delivery.^[^ ^17^ ^]^ However, most oxygen‐carrying materials have long been criticized for their poor biocompatibility.^[^ ^14^ ^]^ Fortunately, perfluorocarbons (PFCs) have been recently used for bolstering exogenous oxygen supplementation into solid tumors, due to their remarkable biocompatibility and high oxygen‐dissolving capacity.^[^ ^18^, ^19^, ^20^, ^21^ ^]^ Weak intermolecular van der Waals interactions and minimal cohesive forces contribute to excellent oxygen dissolubility of PFCs.^[^ ^22^ ^]^ Meanwhile, PFCs also exhibit a conspicuously similar Hildebrandt parameter with oxygen, thereby endowing PFCs‐based nanocarriers with an outstanding oxygen‐carrying capacity in basis of the similarity‐intermiscibility principle.^[^ ^22^ ^]^ Moreover, the inherent chemical inertness property of PFCs safeguards them against potential interactions with endogenous substances within the biological milieu, enabling superior biocompatibility with negligible adverse effects on the body.^[^ ^23^ ^]^ Consequently, PFCs stand as feasible candidates to be developed as excipients for intravenous formulations, even as artificial blood substitutes.^[^ ^22^, ^23^, ^24^ ^]^ Notably, PFCs exhibit a pivotal role in significantly extending the half‐life of reactive oxygen species (ROS), thus underpinning the potential of PFCs‐based nanomedicines to enhance ROS‐mediated therapeutic modalities.^[^ ^24^ ^]^ Additionally, the distinctive physical feature of gas dissolution attributed to PFCs expedites oxygen release when necessitated, in sharp contrast with the covalent binding pattern of oxygen within hemoglobin.^[^ ^22^ ^]^ Nevertheless, we found that oxygen‐carrying ability of conventional PFCs‐based nanomedicines is still far from satisfaction, mainly due to the unsatisfactory PFCs loading efficiency and the retardation effect on oxygen diffusion and dissolution resulted from nanocarrier materials. Moreover, the clinical translation of PFCs‐based oxygen‐supplying strategy is greatly hindered by their highly hydrophobic property and the poor excipient availability of PFCs‐containing polymeric materials.^[^ ^9^, ^10^, ^11^, ^12^ ^]^

In addition to oxygen supply strategy, several clinically available drugs have demonstrated capacity in inhibiting oxygen consumption, such as atovaquone (ATO), metformin, tamoxifen and papaverine.^[^ ^17^ ^]^ Among them, metformin is the singular FDA‐approved drug capable of curtailing the OCR. Nonetheless, its efficacy in OCR reduction remains limited (only 10%–20%) at pharmacologically achievable concentrations.^[^ ^25^, ^26^, ^27^ ^]^ Recently, ATO emerges as a more potent oxygen consumption inhibitor, evoking a pronounced decline in OCR (up to 80%) across multiple tumor cell lines, 3D multicellular tumor spheroids, and xenograft tumor mouse models.^[^ ^25^ ^]^ The effect is attributed to its interference with the mitochondria‐associated oxidative phosphorylation (OXPHOS) metabolic pathway, achieved by the inhibition of the electron transport chain complex III (cytochrome bc1 complex).^[^ ^28^, ^29^, ^30^ ^]^ However, considering the inherently diminished intracellular oxygen levels within solid tumors, the capacity of ATO alone to address tumor hypoxia is greatly constrained in animal models.^[^ ^31^ ^]^ Not only that, the clinical application of ATO is impeded by its unfavorable water‐solubility, rapid bloodstream clearance, and potential off‐target toxicity.^[^ ^32^ ^]^ As previously discussed, we found that hypoxic tumor cells exhibited a rapid surge in cellular OCR when suddenly exposed to abundant oxygen supply. As such, a closed‐loop oxygen supply/consumption modulation strategy for tumor hypoxia alleviation is highly desired, while realizing synchronous and efficient co‐delivery of oxygen and ATO in biological systems remains challenging. Despite the extensive application of biomedical nanotechnology in combination cancer therapy, conventional co‐delivery nano‐vehicles still suffer from low co‐loading efficiency, poor encapsulation stability and inconvenient regulation of dose proportions ascribed to the affinity difference with carrier materials and different drugs.^[^ ^30^, ^31^, ^32^, ^33^, ^34^ ^]^

**Figure 1:**
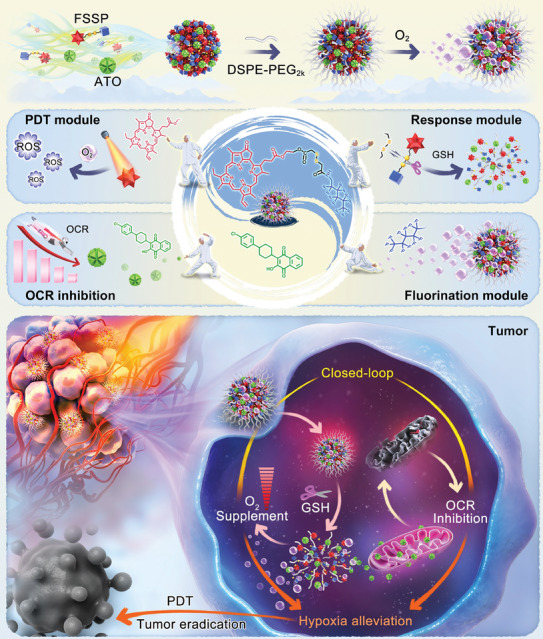
Schematic of carrier‐free oxygen nano‐tank for hypoxia alleviation‐strengthened photodynamic tumor eradication. The nano‐tank was co‐assembled by a fluorinated prodrug (FSSP) of PPa and an oxygen consumption blocker (ATO) without the assistance of any carrier materials. It adeptly replenished oxygen while simultaneously suppressed cellular oxygen consumption for closed‐loop tumor hypoxia alleviation, resulting in satisfactory therapeutic outcomes in vivo.

In the present study, a closed‐loop oxygen nano‐tank was conceived for hypoxia alleviation‐facilitated photodynamic tumor eradication (**Figure** **1**). Specifically, a novel redox‐sensitive prodrug (FSSP) incorporating 1H,1H‐perfluorohexylamine and pyropheophorbide a (PPa) was designed and synthesized, featuring integrated fluorination, responsive and photodynamic modules in one conjugate. The excellent nanoassembly capacity of FSSP prompt itself to spontaneously aggregated into carrier‐free nanoassemblies (NAs). To our excitement, FSSP NAs displayed superior oxygen‐carrying capacity over frequently‐used poly (lactic‐co‐glycolic acid) nanoparticles (PLGA NPs) at equivalent FSSP concentrations. More intriguingly, FSSP was able to co‐assemble with ATO to form binary NAs without the assistance of any carrier materials. Co‐assembly with ATO had negligible impact on the oxygen‐carrying efficiency of FSSP. After the optimization of nanoassembly engineering and oxygen entrapment, an oxygen nano‐tank (FSSPAO NAs) was precisely fabricated at an optimal FSSP/ATO molar ratio of 2:1, with an exceptional oxygen‐dissolving capacity of ≈68 mg L^−1^. FSSP and ATO undertook a dual role as not only vehicles but also as cargos in the nano‐tank, conferring FSSPAO NAs with synchronous co‐delivery feature and high FSSP/ATO co‐loading capability. As shown in Figure 1, the fluorination module in FSSP not only facilitated favorable co‐assembly performance with ATO, but also imparted the nanoassembly with a remarkable prowess in oxygen carrying. Moreover, the nano‐tank displayed a tumor‐specific disassembly feature elicited by the glutathione (GSH)‐triggered cleavage of disulfide bonds in FSSP, which not only effectively relieved the aggregation‐caused quenching (ACQ) dilemma of PPa, but also facilitated tumor‐specific release of ATO. Finally, 4T1 breast and CT26 colon tumor‐bearing mouse models were established to evaluate in vivo antitumor activity of the nano‐tank. Notably, PDT is ideally suited for the treatment of breast cancer, which is typically malignant tumor located in superficial part of the body.^[^ ^33^ ^]^ Meanwhile, PDT is also widely investigated to manage colon cancer in an optical instrument‐intervented way.^[^ ^34^ ^]^ Moreover, as common solid tumors in clinic, both breast cancer and colon cancer are usually characterized with severe hypoxia microenvironments.^[^ ^17^ ^]^ As anticipated, FSSPAO NAs yielded a hypoxia alleviation window and potentiated PDT under laser irradiation in 4T1 breast and CT26 colon tumor‐bearing mouse models. To our knowledge, this is the first attempt to develop a credible oxygen nano‐tank based on modular prodrug design and carrier‐free prodrug/drug nanoassembly technique. This study drives a conceptual step forward in the realm of hypoxia relief‐potentiated anticancer modality.

## 2 Results and Discussion

### 2.1 Modular Design of Fluoridized Prodrugs

This project started with an interesting attempt to modularly design functional prodrug (Figure 1), which consisted of oxygen‐carrying/nanoassembly module (fluorination chain), tumor stimuli‐responsive module (disulfide bond) and photodynamic module (PPa). Specifically, we proposed that the incorporation of the fluorination module would not only foster molecular nanoassembly, but also endow the nanoassembly with the capability of proficient oxygen‐carrying delivery. Meanwhile, the response module inserted within the prodrug was expected to address the ACQ dilemma of PPa following the cleavage of disulfide bonds in the presence of glutathione (GSH) overproduced in tumor cells. Together, the synergistic interplay between the fluorination module and tumor stimuli‐responsive module would contribute to magnifying the therapeutic outcomes of photodynamic module (PPa).

**Figure 2:**
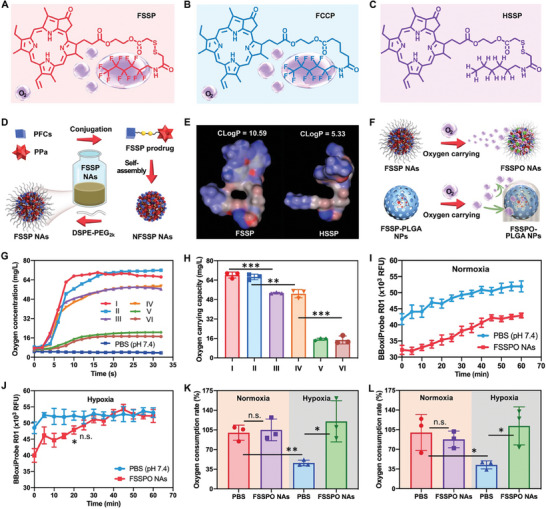
Nanoassembly characteristics, oxygen‐loading capacity, and cellular oxygen consumption rates. A–C) Chemical structures of prodrugs. D) Schematic representation of the self‐assembly process. E) CLogP of FSSP and HSSP. F) Schematic representation of oxygen‐loading features of FSSPO NAs and FSSPO‐PLGA NPs. G) Oxygen concentration changes after addition of different formulations into the deoxygenated water under ultrasonic conditions. The I, II, III, IV, V and VI represent FSSPO NAs, FCCPO NAs, FSSPO‐PLGA NPs, FCCPO‐PLGA NPs, HSSPO NAs and HSSPO‐PLGA NPs, respectively. H) Quantitative analysis of oxygen‐loading capacity (n = 3). I) Fluorescence of the oxygen probe in 4T1 cells incubated with PBS (pH 7.4) or FSSPO NAs under normoxic conditions within 60 min (n = 3). J) Fluorescence of the oxygen probe in 4T1 cells incubated with PBS (pH 7.4) or FSSPO NAs under hypoxic conditions within 60 min (n = 3). K) Quantitative analysis of oxygen consumption rates of 4T1 cells incubated with PBS (pH 7.4) or FSSPO NAs under normoxic or hypoxic conditions at 60 min (n = 3). L) Quantitative analysis of oxygen consumption rates of CT26 cells incubated with PBS (pH 7.4) or FSSPO NAs under normoxic or hypoxic conditions at 60 min (n = 3).

To validate our hypothesis, a modularly multifunctional prodrug was designed and synthesized (**Figure** **2**A,C; Figure S1, Supporting Information). As illustrated in Figure S1 (Supporting Information), an important chemical intermediate of the photosensitizer with hydroxyl group (PPa‐EG) was initially obtained by conjugating PPa with ethylene glycol (EG). Subsequently, the fluoridized prodrug (FSSP) was synthesized by coupling 1H,1H‐perfluorohexylamine to PPa‐EG via a disulfide bond as linkage (Figure 2A; Figure S1, Supporting Information). In parallel, PPa‐EG was conjugated to 1H,1H‐perfluorohexylamine using an equiatomic carbon chain leading to the non‐sensitive control prodrug (FCCP, Figure 2B). Moreover, a non‐fluoridized prodrug (HSSP) was synthesized following a similar synthesis route as FSSP, with the exception that 1H,1H‐perfluorohexylamine was substituted by hexylamine (Figure 2C; Figure S1, Supporting Information). The successful synthesis of these PPa prodrugs was thoroughly characterized and confirmed through mass spectrometry (MS) and proton nuclear magnetic resonance (^1^H NMR) analysis (Figure S2, Supporting Information), respectively. The purity of FSSP, FCCP and HSSP was more than 98.95% (Figure S3, Supporting Information). Furthermore, the ultraviolet and fluorescence spectra of PPa, FSSP, FCCP and HSSP were obtained at an equivalent PPa concentration. As depicted in Figure S4 (Supporting Information), no substantial alterations were observed in the peak position and strength of both the ultraviolet and fluorescence spectra, suggesting the negligible influence of chemical modifications on the optical features of PPa.

### 2.2 Self‐Assembly of PPa Prodrugs

As previously elucidated, the fluoridized prodrugs were anticipated to exhibit advantageous attributes in terms of nanoassembly and oxygen‐carrying capabilities, due to the inherent hydrophobicity and exceptional oxygen‐dissolving potential of the fluorination module. To achieve this, a one‐step nanoprecipitation technique was adeptly employed to fabricate prodrug nanoassembly (Figure 2D). As depicted in Figure S5 (Supporting Information), an interesting nanoassembly phenomenon spontaneously manifested upon the dispersion of FSSP, FCCP or HSSP in deionized water without any carrier materials. Then, 1, 2‐distearoyl‐sn‐glycero‐3‐phosphoethanolamine‐N‐[methoxy(polyethyleneglycol)−2000] (DSPE‐PEG_2k_) was utilized to implement PEGylation decoration on the NAs. As shown in Table S1 (Supporting Information), the particle size of both FSSP NAs and FCCP NAs was ≈70 nm, while concurrently exhibiting negatively Zeta potentials (≈ −23 to ‐24 mV). It is noteworthy that the fluoridized prodrug NAs (FSSP NAs and FCCP NAs) demonstrated a relatively smaller particle size in comparison to the non‐fluoridized HSSP NAs (≈80 nm). These results substantiated our hypothesis that the fluoridized side chain with stronger hydrophobicity endowed FSSP and FCCP with more optimal nanoassembly performance, forming more compact nanostructures.^[^ ^35^, ^36^ ^]^ The contribution of hydrophobic fluorination chain to nanoassembly was further underscored in Figure 2E, which highlights the calculated LogP (CLogP) values of 10.59 for FSSP and 5.33 for HSSP, respectively.

### 2.3 Oxygen‐Carrying Capacity of Fluoridized Prodrug NAs

The remarkable nanoassembly feature of fluoridized prodrugs prompted us to embark on their oxygen‐carrying capacity. Notably, one of the pivotal virtues inherent to carrier‐free prodrug‐assembled nanomedicines lies in impressively high drug loading capacity. It is noteworthy that FSSP NAs not only had a high loading rate of PPa (≈41.76%), but also enabled a high loading rate (≈23.38%) of 1H,1H‐perfluorohexylamine (the fluorination module). This exceptional fluorine loading capacity of fluoridized prodrug NAs would confer oxygen‐carrying performance advantages over nanomaterial‐based nanocarriers (Figure 2F). To establish a comparative analysis of oxygen‐carrying capacity with carrier material‐based nanosystems, the frequently‐used PLGA nanoparticles (NPs) were fabricated to encapsulate the same prodrugs (FSSP, FCCP or HSSP), respectively. Remarkably, FSSP‐PLGA NPs exhibited a considerably lower fluorine loading rate (≈2.30%) than our carrier‐free FSSP NAs (≈23.38%). Such a stark difference propelled us to engage in a comprehensive comparison of the oxygen‐carrying attributes between the prodrug NAs and PLGA NPs, employing an equivalent concentration of 1H,1H‐perfluorohexylamine. The oxygen‐carrying prodrug NAs were named FSSPO NAs, FCCPO NAs and HSSPO NAs, while their PLGA NPs were named FSSPO‐PLGA NPs, FCCPO‐PLGA NPs and HSSPO‐PLGA NPs, respectively. After placing the oxygen‐saturated prodrug NAs and PLGA NPs into the deoxygenated water, with real‐time monitoring of oxygen concentration changes under ultrasonic conditions. As illustrated in Figure 2G,H, carrier‐free fluoridized prodrug NAs (FSSPO NAs and FCCPO NAs) exhibited markedly superior oxygen carrying efficiency (≈68 mg L^−1^) compared to their the corresponding FSSPO‐PLGA NPs and FCCPO‐PLGA NPs counterparts (≈52 mg L^−1^). The minimal variations fluctuations in oxygen concentration‐time curves were observed for both HSSPO NAs and HSSPO‐PLGA NPs could be attributed to modest oxygen adsorption by colloidal particles (Figure 2G,H). These findings robustly underscored the oxygen‐carrying potential conferred by the fluorination module in fluoridized prodrug NAs and PLGA NPs. Significant, carrier‐free prodrug NAs demonstrated distinct advantages over PLGA‐based oxygen nanocarriers under identical conditions, owing to the retardation effect of heavy nanocarrier materials on oxygen diffusion and dissolution in PLGA NPs (Figure 2F).

### 2.4 Abnormal Cellular OCR Elevation under Hypoxia

As earlier mentioned, exogenous oxygen supply encounters a substantial counteraction due to the marked escalation in cellular oxygen consumption under hypoxic conditions. This phenomenon arises from the compensatory and retaliatory oxygen consumption by hypoxic tumor cells when suddenly exposed to abundant oxygen supply. To substantiate this hypothesis, we conducted real‐time monitoring of the relative oxygen consumption rates of 4T1 cells treated with or without FSSPO NAs under normoxic or hypoxic conditions within 60 min, respectively. A BBoxiProbe R01 kit was used to evaluate the cellular oxygen consumption by real‐time determination of fluorescence intensity, wherein stronger fluorescence signals represent the lower cellular oxygen content. The real‐time oxygen consumption of 4T1 cells treated with PBS (pH 7.4) or FSSPO NAs under normoxic and hypoxic conditions within 60 min was shown in Figure 2I,J, respectively. Moreover, the oxygen consumption rates (OCR) of 4T1 cells (Figure 2K) and CT26 cells (Figure 2L) were calculated by the fluorescence intensity difference between 60 and 0 min under normoxic and hypoxic conditions. As shown in Figure 2I,J, there was a wide RFU gap between PBS‐treated and FSSPO NAs‐treated cells at 0 min, suggesting the ability of FSSPO NAs to supply oxygen. Notably, although there was a difference in fluorescence intensity, no significant difference was observed in the OCR between PBS‐treated and FSSPO NAs‐treated cells under normoxic conditions (Figure 2I). This result showed that although FSSPO NAs significantly increased cellular oxygen content, it had little impact on the OCR of 4T1 cells under normoxic conditions. By contrast, the OCR of NAs‐treated cells was elevated significantly when compared to that of PBS‐treated cells under hypoxic conditions (Figure 2J), suggesting that the OCR of hypoxic tumor cells significantly increased when exposed to oxygen supply by FSSPO NAs. As shown in Figure 2K, there was scarcely any significant difference in the OCR between 4T1 cells exposed to FSSPO NAs and PBS‐treated cells under normoxic conditions, while the oxygen consumption in 4T1 cells treated with FSSPO NAs was notably elevated compared to untreated cells under hypoxic conditions. Similar trends were found in CT26 cells (Figure 2L; Figure S6, Supporting Information). These findings confirmed the excellent oxygen replenishment ability of FSSPO NAs. Unfortunately, oxygen supply alone did not seem to effectively relieve hypoxia, due to the retaliatory oxygen consumption of hypoxic tumor cells.

### 2.5 Molecular Engineering of a Closed‐Loop Oxygen Nano‐Tank

**Figure 3:**
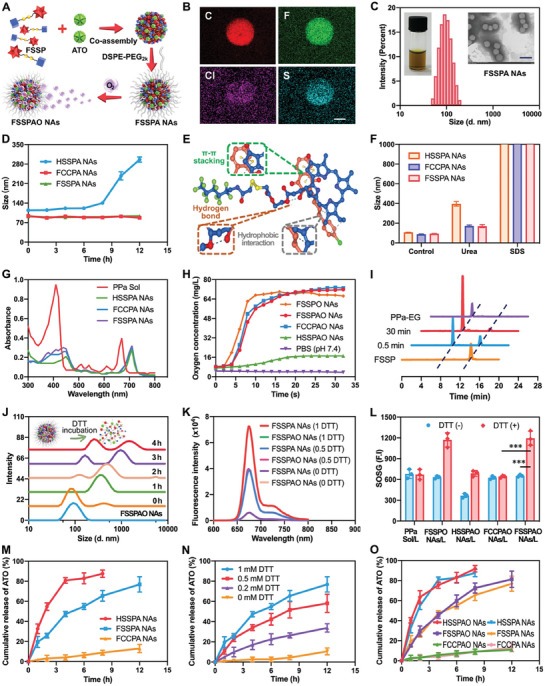
Fabrication and characterization of the oxygen nano‐tank. A) Schematic representation of FSSP/ATO co‐assembly. B) EDS element mapping images of FSSPA NAs (scale bar represents 50 nm). C) Appearance photo, particle size distribution profile and TEM image of FSSPA NAs (scale bar represents 200 nm). D) Colloidal stability of three co‐assembled NAs incubated in PBS (pH 7.4) containing 10% FBS (n = 3). E) Molecular docking simulation results of FSSP/ATO co‐assembly. F) Particle size changes of three co‐assembled NAs treated with urea (200 mM) and SDS (200 mM). G) Ultraviolet absorption spectra of PPa Sol and three co‐assembled NAs at 300–800 nm wavelength. H) Oxygen concentration changes after addition of different formulations into the deoxygenated water under ultrasonic conditions. I) DTT‐triggered degradation of FSSP into PPa‐EG at different incubation time using HPLC analysis. J) Particle size of FSSPAO NAs in the presence of DTT (1 mM). K) Excitation spectral changes of FSSPA NAs and FSSPAO NAs incubated in media containing various concentrations (0, 0.5 and 1 mM) of DTT for 4 h. L) SOSG fluorescence signals of PPa Sol, FSSPO NAs, HSSPAO NAs, FCCPAO NAs and FSSPAO NAs incubated in media containing DTT (1 mM) under 660 nm laser irradiation (20 mW cm^−2^, n = 3). The “L” represents laser irradiation (660 nm, 20 mW cm^−2^, 5 min). M) ATO release profiles from FSSPA NAs, FCCPA NAs and HSSPA NAs in the presence of DTT (1 mM, n = 3). N) ATO release profiles from FSSPA NAs in the presence of various concentrations of DTT (n = 3). O) ATO release profiles from NAs in the presence of DTT (1 mM, n = 3).

As previously observed, the alleviation of tumor hypoxia through exogenous oxygen supply can be significantly hampered by the abnormal elevation in cellular OCR under hypoxic conditions (Figure 2I–L; Figure S6, Supporting Information). To address the challenge, we proposed to devise a comprehensive oxygen‐enriching approach that would encompass both supply and consumption facets, ultimately bringing up a hypoxia alleviation window to enhance PPa‐mediated PDT. Fortunately, an interesting nanoassembly phenomenon between PPa prodrugs (FSSP, FCCP and HSSP) and an oxygen consumption inhibitor (ATO) was serendipitously discovered during preliminary experimental endeavors. As mentioned earlier, ATO exhibited a remarkable capacity to increase cellular oxygen content within tumor cells by inhibiting mitochondrial respiration. Based on these intriguing findings, we intended to develop an oxygen nano‐tank self‐assembled from FSSP and ATO, which was expected to relieve tumor hypoxia through a closed‐loop mechanism. Notably, ATO was found to be inept in self‐assembling into NAs (Figure S7, Supporting Information), while it was able to readily co‐assemble with FSSP into uniform nanostructures without the need for additional carrier materials (**Figure** **3**A). A very small amount of DSPE‐PEG_2k_ was used as modifier to increase colloidal stability and prolong circulation time in the blood after intravenous administration. To optimize the proportion of DSPE‐PEG_2k_, different amount of DSPE‐PEG_2k_ was applied to prepared PEGylated FSSP/ATO NAs, respectively. As shown in Table S2 (Supporting Information), the FSSP/ATO NAs with 20 wt.% and 30 wt.% DSPE‐PEG_2k_ demonstrated a smaller particle size (≈90 nm) in comparison to that of FSSP/ATO NAs with 10 wt.% DSPE‐PEG_2k_ (≈110 nm). Comprehensively considering the drug loading capacity and nanoassembly performance, 20 wt.% of DSPE‐PEG_2k_ was ultimately employed to fabricate the PEGylated hybrid nanomedicines, named as FSSPA NAs. To further optimize the conditions of nanoassembly engineering, a series of PEGylated FSSP/ATO hybrid NAs were prepared at various molar ratios, including 4:1, 3:1, 2:1, 1:1, 1:2, 1:3 and 1:4 (FSSP/ATO). As outlined in Table S3 (Supporting Information), the hybrid NAs (FSSPA NAs) formed at a molar ratio of 2:1 (FSSP/ATO) emerged as the optimal formulation, exhibiting desirable particle size and colloidal uniformity. It is noteworthy that FSSPA NAs not only had a high loading rate of ATO (≈12.17%), but also enabled a high loading rate of FSSP (≈67.83%). Further validation of the successful co‐assembly between FSSP and ATO was accomplished through elemental mapping utilizing scanning electron microscopy (SEM) coupled with energy dispersive X‐ray spectrometry (EDS). Figure 3B and Table S5 (Supporting Information) visually depicted the elemental mapping results, which unequivocally indicated the co‐localization of FSSP and ATO within the nanostructures.

Subsequently, PEGylated FCCP/ATO NAs and HSSP/ATO NAs were fabricated at an identical molar ratio of 2:1 (prodrug/ATO) as the non‐sensitive and non‐fluoridized control NAs, respectively. As depicted in Figure 3C, Figure S8A and B and Table S4 (Supporting Information), all these prodrug/ATO nano‐formulations (FSSPA NAs, FCCPA NAs and HSSPA NAs) exhibited consistently spherical morphology (80–110 nm) coupled with desirable colloidal uniformity (PDI < 0.2) and exhibited negative Zeta potentials (≈ ‐25 mV). The incorporation of negative charges in nanomedicines holds promise in favorable pharmacokinetics by resisting abundant plasma protein adsorption following intravenous administration.^[^ ^37^, ^38^, ^39^, ^40^ ^]^ Significantly, the fluoridized hybrid NAs (FSSPA NAs and FCCPA NAs) demonstrated notably smaller particle sizes (≈90 nm) than that of the non‐fluoridized HSSPA NAs (≈107 nm), a trend consistent with the outcomes of self‐assembled prodrug NAs (Table S1, Supporting Information). The same principles underlying the co‐assembled NAs elucidated that the pronounced hydrophobicity of 1H,1H‐perfluorohexylamine contributed to favorable nanoassembly features of fluoridized prodrugs (Figure 2E), thus aggregating in more compact nanostructures (Table S3, Supporting Information). Due to the relatively inferior nanoassembly characteristics, HSSPA NAs exposed to PBS (pH 7.4) containing 10% FBS showed poor colloidal stability with remarkable increase of particle size and PDI (Figure 3D and S8C). In stark contrast, both FSSPA NAs and FCCPA NAs displayed favorable colloidal stability within 12 h under the same conditions (Figure 3D; Figure S8C, Supporting Information). In addition, FSSPA NAs showed favorable colloidal stability under PBS (pH 5.6, 6.4 and 7.4) incubation and laser irradiation (Figure S8D and E, Supporting Information). These results suggested that the fluoridized prodrugs (FSSP and FCCP) could serve as an eligible nanoassembly partner for ATO.

We then explore the underlying nanoassembly mechanisms of FSSP and ATO. To illuminate the intermolecular interactions and forces within the hybrid NAs of FSSP and ATO, we employed computational modeling and molecular docking simulations. As illustrated in Figure 3E, a variety of intermolecular forces were found in FSSPA NAs, including hydrogen bonds, hydrophobic forces and π‐π stacking interactions. Among them, the photosensitive moiety (PPa) mainly provided hydrophobic forces and π–π stacking interactions. These results demonstrated that the nanoassembly ability of FSSP should be is a composite effect of the hydrophobic side chain and the photosensitive moiety in the prodrug. To authenticate the presence of these intermolecular interactions/forces, we further conducted intermolecular force failure experiments.^[^ ^41^ ^]^ The NAs were subjected to incubation with sodium dodecyl sulfate (SDS) and urea, aimed at elucidating the primary nanoassembly driving forces. As illustrated in Figure 3F, the particle size of FSSPA NAs, FCCPA NAs and HSSPA NAs exhibited a significant increase in the presence of SDS, underscoring the pivotal role of hydrophobic interactions during the nanoassembly process. Furthermore, upon incubation with urea, the mean diameter of FSSPA NAs, FCCPA NAs and HSSPA NAs also displayed slight increments compared to the control group (Figure 3F), suggesting the considerable contribution of hydrogen bonds. Notably, a more pronounced change in particle size was observed in HSSPA NAs compared to FSSPA NAs and FCCPA NAs when exposed to urea (Figure 3F), indicating the greater significance of hydrogen bonds in driving the co‐assembly of HSSP and ATO. As an additional affirmation of these interactions, the ultraviolet spectra of FSSPA NAs, FCCPA NAs and HSSPA NAs displayed a noticeable red shifted when compared to PPa solution (Sol), confirming the existence of the π–π stacking interactions in these hybrid NAs (Figure 3G). These results collectively provided compelling evidence that multiple interactions and forces collaboratively propelled the nanoassembly of PPa prodrugs and ATO.

We then focused on their oxygen‐carrying capabilities, particularly focusing on the influence of ATO on oxygen‐carrying capacity. These oxygen‐carrying prodrug/ATO hybrid NAs were designated as FSSPAO NAs, FCCPAO NAs and HSSPAO NAs, respectively. As illustrated in Figure 3H, the fluorinated hybrid NAs (FSSPAO NAs and FCCPAO NAs) demonstrated a remarkably consistent absolute oxygen‐carrying capacity (≈68 mg L^−1^) with the self‐assembled NAs of fluorinated prodrugs (FSSPO NAs and FCCPO NAs) (Figure 2G). As anticipated, HSSPAO NAs demonstrated almost negligible oxygen‐carrying capacity (Figure 3H). These findings implied that co‐assembly with ATO had minimal impact on the oxygen‐carrying capacity of fluorinated prodrugs. The excellent oxygen‐carrying capacity exhibited by FSSPAO NAs fulfilled our expectations that was qualified to tackle tumor hypoxia through FSSP‐based oxygen supply and ATO‐mediated suppression of cellular oxygen consumption. Additionally, we also investigated the effect of oxygen loading on the colloidal stability of NAs. As indicated in Figure S8F (Supporting Information), both FSSPA NAs and FSSPAO NAs maintained good colloidal stability within 12 h when incubated with PBS (pH 7.4) containing 10% FBS. Given its dual functionality of oxygen supply and consumption inhibition, we termed this uniquely engineered nanoassembly as the oxygen nano‐tank (FSSPAO NAs). Significant, such a nano‐tank is poised to effectively address the challenges arising from the compensatory OCR increase of hypoxic tumor cells following exogenous oxygen supply.

### 2.6 Redox‐Responsive Prodrug Activation‐Facilitated ACQ Relief and ATO Release

The rational design of prodrugs not only addresses the suboptimal physicochemical properties of drugs through strategic chemical modifications, but also imparts the potential for drug nanoassembly in certain cases.^[^ ^37^, ^38^ ^]^ Moreover, an equally significant advantage of prodrug strategy lies in its capacity for on‐demand activation, facilitated by the incorporation of suitable chemical linkers in the conjugates, resulting in desired therapeutic outcomes with reduced toxicity and side effects. Notably, the structural breakdown of self‐engineered prodrug NAs occurs in tandem with the disintegration of conjugates.^[^ ^37^, ^38^ ^]^ Such phenomenon significantly impacted tumor‐specific drug delivery and on‐demand drug release, thus amplifying therapeutic efficacy and safety. In addition, the cytosolic GSH concentration in tumor cells is up to 1–10 mM, which is ≈7 times higher than that in normal cells.^[^ ^42^, ^43^ ^]^ In the present study, we envisaged that the redox‐responsive activation of FSSP conjugate in the hybrid NAs would not only efficiently handle the ACQ dilemma of PPa, but also facilitate tumor‐specific ATO release following the collapse of FSSPAO NAs triggered by GSH overproduced in tumor cells.

To explore the reductive responsiveness of the hybrid NAs, we employed dithiothreitol (DTT), a widely utilized GSH surrogate known for its enhanced chemical stability. As demonstrated in Figure 3I, the concentration of FSSP exhibited gradual reduction over time in the presence of DTT. In contrast, the non‐sensitive conjugate (FCCP) remained largely unchanged under identical conditions (Figure S9, Supporting Information). Given the pivotal role of the fluorinated side chain (1H,1H‐perfluorohexylamine) in the nanoassembly process, the activation of the redox‐responsive prodrug subsequently triggered the disintegration of nano‐tank (FSSPAO NAs, Figure 3J), thereby expectedly mitigating the ACQ effect and facilitating ATO release. To validate our hypothesis, the fluorescence spectra of FSSPAO NAs, FSSPA NAs, FCCPA NAs, and HSSPA NAs were monitored upon incubation in media containing various concentrations of DTT. As depicted in Figures 3K and S10 (Supporting Information), the fluorescence intensity of formulations containing disulfide bonds (FSSPAO NAs, FSSPA NAs and HSSPA NAs) substantially intensified in a concentration‐dependent manner upon exposure to DTT. This phenomenon could be ascribed to the triggered collapse of redox‐responsive NAs triggered by DTT (Figure 3J). By contrast, the fluorescence of FCCPA NAs was significantly quenched from beginning to end, even in the presence of 1 mM DTT for 4 h (Figure S10, Supporting Information). Moreover, minimal disparity was observed disparity between FSSPAO NAs and FSSPA NAs (Figure 3K; Figure S10, Supporting Information), suggesting the negligible effect of oxygen on the reductive responsiveness of FSSP. These results validated the pivotal role of redox stimuli‐triggered prodrug activation in effectively mitigating the ACQ dilemma.

In addition to fluorescence quenching, photodynamic photosensitizers confined within nanocarriers often exhibit a sharp reduction in singlet oxygen production under laser irradiation.^[^ ^2^ ^]^ To investigate singlet oxygen generation from the NAs, we employed a singlet oxygen sensor green (SOSG) reagent kit. As depicted in Figure S11 (Supporting Information), the SOSG fluorescence signals emitted by the oxygen‐carrying NAs (FCCPAO NAs and FSSPAO NAs) far significantly exceeded those originating from oxygen‐free NAs (FSSPA NAs, HSSPAO NAs and FCCPA NAs) under laser irradiation, affirming the important role of oxygen in singlet oxygen generation. Subsequently, we delved into the impact of DTT on the singlet oxygen generation of oxygen‐carrying NAs. As shown in Figure 3L, the introduction of DTT had negligible effects on the singlet oxygen generation of PPa. Significant, the fluorescence intensity of NAs containing disulfide bond (FSSPAO NAs, FSSPO NAs and HSSPAO NAs) was dramatically amplified in the presence of DTT (1 mM) under laser irradiation (Figure 3L). In contrast, FCCPAO NAs displayed no appreciable alteration under the same conditions (Figure 3L). Remarkably, the total singlet oxygen yield of FSSPAO NAs incubated in DTT (1 mM) even showed distinct advantage over PPa Sol at an equivalent PPa concentration (Figure 3L), demonstrating the remarkable photodynamic conversion efficiency of FSSPAO NAs. The favorable singlet oxygen generation of FSSPAO NAs and FSSPO NAs incubated with DTT should be attributed to the composite effect of ACQ relief and oxygen supply. Collectively, these findings firmly supported the qualification of such a bidirectional oxygen nano‐tank as a potent PDT nanomedicine.

As shown in Figure 3J, the breakage of FSSP triggered by DTT led to the disintegration of FSSPAO NAs, consequently facilitating rapid ATO release from the NAs due to the weakening or disappearance of intermolecular interactions and forces between FSSP and ATO. To investigate the in vitro ATO release patterns, a minor quantity of tetrahydrofuran (20%, v/v) was added into PBS (pH 7.4) to solubilize the hydrophobic ATO aqueous in release media. As depicted in Figures 3M,N and S12 (Supporting Information), both FSSPA NAs and HSSPA NAs exhibited redox‐sensitive ATO release behaviors in a DTT concentration‐dependent manner. In contrast, the non‐sensitive FCCPA NAs demonstrated an extremely sluggish ATO release, accounting for less than 20% release within 12 h (Figure 3M). Notably, HSSPA NAs displayed a relatively faster drug release rate compared to FSSPA NAs under the same conditions (Figure 3M; Figure S12, Supporting Information), potentially due to the poor stability of non‐fluorinated hybrid NAs (Figure 3D). Furthermore, the presence of oxygen exerted minimal influence on ATO release from the NAs (Figure 3O). these results comprehensively substantiate our proposition that this a redox‐responsive oxygen nano‐tank (FSSPAO NAs) not only effectively mitigates the ACQ obstacle of PPa, but also promoted on‐demand ATO release from the nano‐tank.

### 2.7 Cellular Uptake

**Figure 4:**
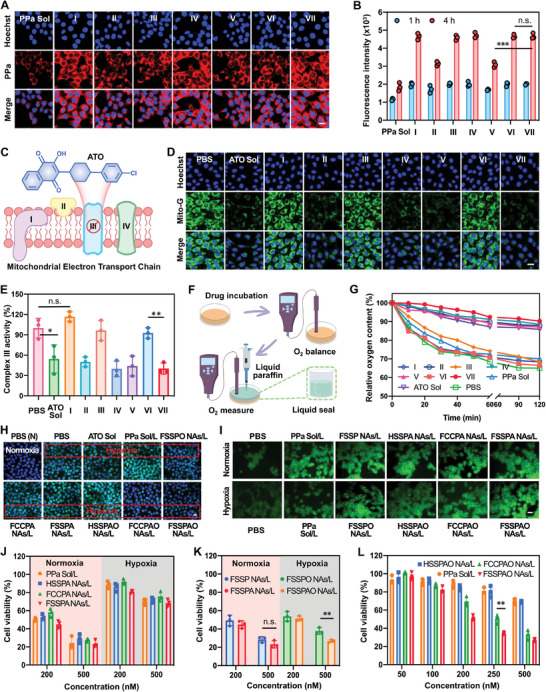
Cellular uptake, hypoxia alleviation and in vitro antitumor effect of NAs. A) Cellular uptake in 4T1 cells at 4 h (Scale bar: 10 µm). B) Quantitative analysis of cellular uptake in 4T1 cells via a multimode microplate reader (Thermo Scientific, USA). C) Chemical structure of ATO and its mechanism on reducing oxygen consumption via inhibiting the mitochondrial electron transport chain complex III. D) CLSM images of 4T1 cells treated with different formulations, stained by Mito‐G (Scale bar: 10 µm). E) Relative activity of mitochondrial complex III in 4T1 cells treated with different formulations. F) Schematic representation of real‐time cellular oxygen consumption monitoring. G) Relative oxygen content of 4T1 cells receiving different formulations. H) Immunofluorescence images of HIF‐1α proteins in 4T1 cells incubated with PBS or NAs (Scale bar: 20 µm). I) Cellular ROS generation in 4T1 cells after incubation with different formulations (Scale bar: 20 µm). J) Cytotoxicity of different formulations in 4T1 cells under normoxic and hypoxic conditions. K) Cytotoxicity of FSSP NAs/L and FSSPA NAs/L in 4T1 cells under normoxic conditions, and cytotoxicity of FSSPO NAs/L and FSSPAO NAs/L in 4T1 cells under hypoxic conditions. L) Cytotoxicity of different formulations in 4T1 cells under hypoxic conditions. The I, II, III, IV, V, VI and VII represent FSSPO NAs, HSSPA NAs, FCCPA NAs, FSSPA NAs, HSSPAO NAs, FCCPAO NAs and FSSPAO NAs, respectively. The “L” represents laser irradiation (660 nm, 20 mW cm^−2^, 5 min).

To unveil cellular uptake efficiency of the nano‐tank, the intracellular fluorescence intensity of PPa in 4T1 and CT26 cells was detected following incubation with different formulations. As depicted in **Figure** **4**A and Figures S13 and S14A–C (Supporting Information), the cellular internalization of both PPa Sol and NAs exhibited a time‐dependent pattern. Notably, the cellular fluorescence intensity of oxygen‐carrying NAs (HSSPAO NAs, FCCPAO NAs, and FSSPAO NAs) closely resembled that of oxygen‐free NAs (HSSPA NAs, FCCPA NAs, and FSSPA NAs), suggesting that the presence of oxygen had minimal influence on cellular uptake (Figure 4A). Furthermore, FSSPAO NAs and FSSPO NAs exhibited comparable cellular uptake efficiency at both 1 and 4 h (Figure 4A), implying that the inclusion of ATO had marginal impact on cellular uptake efficiency. Importantly, the cellular fluorescence intensity of disulfide bond‐containing NAs (FSSPAO NAs, FSSPA NAs and FSSPO NAs) greatly exceeded that of non‐fluorinated NAs (HSSPAO NAs and HSSPO NAs) and non‐sensitive NAs (FCCPAO NAs and FCCPA NAs) under the same conditions, particularly at 4 h post incubation (Figure 4A and Figures S13 and S14A–C, Supporting Information). The reduced cellular uptake of non‐fluorinated NAs should be attributed to their inferior stability in the presence of salts (PBS) and FBS (Figure 3D). In contrast, the enhanced cellular uptake of disulfide bond‐containing NAs stemmed to not only from their improved colloidal stability, but also from the alleviated ACQ effect in the presence of GSH overproduced in tumor cells (Figure 3D,K). The comparatively low cellular fluorescence intensity of non‐sensitive NAs might be ascribed to the ACQ dilemma of PPa. To validate this observation, the actual fluorescence intensity of PPa Sol and NAs after cell disruption and nanostructure destruction was quantitatively determined using a multimode microplate reader (Thermo Scientific, USA). Under such conditions, no significant difference was observed between non‐sensitive NAs (FCCPAO NAs and FCCPA NAs) and disulfide bond‐containing FSSPAO NAs, FSSPA NAs and FSSPO NAs (Figure 4B; Figure S14D, Supporting Information). The remarkably similar nanostructures, particle sizes and Zeta potentials of fluorinated prodrug NAs resulted in closely aligned cellular uptake trends (Figure 3C; Figure S8A,B, Supporting Information), while the ACQ effect of PPa trapped in non‐sensitive NAs engendered a misleading impression of inferior cellular uptake (Figure 4A). These findings further indicated that tumor stimuli‐responsive prodrug NAs enabled to effectively address the challenge of ACQ dilemma.

### 2.8 Suppression of Cellular Oxygen Consumption

The exceptional oxygen‐carrying capacity of the nano‐tank (Figure 3H) motivated us to explore ATO‐mediated cellular oxygen consumption (Figure 4C). Mitochondrial marker Mito‐G was employed to visualize mitochondria. As shown in Figures 4D and S15 (Supporting Information), robust green fluorescence was evident in 4T1 and CT26 cells treated with ATO‐free formulations (PBS or FSSPO NAs) and non‐sensitive FCCPAO NAs. In contrast, the green fluorescence in 4T1 and CT26 cells exposed to ATO Sol, HSSPAO NAs, and FSSPAO NAs characterized by redox‐responsive ATO release was significantly diminished, indicative of substantial mitochondrial impairment (Figure 4D; Figure S15, Supporting Information). Notably, the limited impact of FCCPAO NAs on mitochondria stemmed from their gradual ATO release (Figures 3M and  4D). Furthermore, oxygen‐free formulations (HSSPA NAs and FSSPA NAs) exhibited comparable fluorescence intensity with oxygen‐loaded counterparts (HSSPAO NAs and FSSPAO NAs), suggesting that oxygen had negligible influence on ATO‐induced mitochondria damage (Figure 4D). As previously elucidated, ATO curtailed cellular OCR by inhibiting the activity of mitochondrial electron transport chain (ETC) complex III (cytochrome *bc*_1_ complex). To verify this mechanism, the activity of mitochondrial complex III in both 4T1 and CT26 cells exposed to different formulations was evaluated. As depicted in Figures 4E and S16 (Supporting Information), ATO Sol, HSSPAO NAs and FSSPAO NAs significantly suppressed the activity of mitochondrial complex III activity in comparison to PBS, FSSPO NAs and FCCPAO NAs (Figure 4E; Figure S16, Supporting Information). Similarly, oxygen delivery had minimal impact on mitochondrial complex III, which was consistent with the results observations in Figure 4D.

Next, the real‐time oxygen content in cell culture medium was monitored utilizing a dissolved oxygen meter under oxygen‐isolated conditions (Figure 4F). As illustrated in Figures 4G and S17A (Supporting Information), the oxygen content in both 4T1 and CT26 cells treated with PBS and non‐responsive FCCPAO NAs rapidly declined in a time‐dependent manner, suggesting continuous oxygen consumption by tumor cells. In contrast, the oxygen content of cells treated with ATO‐containing formulations (FSSPAO NAs, HSSPAO NAs, FSSPA NAs, HSSPA NAs and ATO Sol) remained ≈at 90% within 2 h, demonstrating that ATO Sol and redox‐sensitive ATO‐containing NAs effectively inhibited intracellular oxygen consumption (Figure 4G; Figure S17A, Supporting Information). Importantly, despite the initially higher oxygen content in tumor cells receiving oxygen‐carrying NAs (Figure S17B, Supporting Information), there was minimal difference in the relative oxygen content changes between oxygen‐carrying NAs (HSSPAO NAs, FCCPAO NAs and FSSPAO NAs) and oxygen‐free NAs (HSSPA NAs, FCCPA NAs and FSSPA NAs), suggesting the negligible effect of oxygen on ATO‐mediated OCR inhibition (Figure 4G; Figure S17A, Supporting Information).

### 2.9 Cellular HIF‐1α Expression

We next conducted immunofluorescence staining and western blot assay to assess the expression of hypoxia inducible factor (HIF‐1α) in both 4T1 and CT26 cells treated with different formulations. As shown in Figure 4H and Figure S18 (Supporting Information), minimal HIF‐1α expression was observed in 4T1 and CT26 cells treated with PBS (pH 7.4) under normoxic conditions. In contrast, obvious immunofluorescence indicative of HIF‐1α overexpression was observed in both 4T1 and CT26 cells treated with PBS (pH 7.4) under hypoxic conditions. Among these formulations, HSSPAO NAs without oxygen‐carrying ability failed to downregulate the HIF‐1α overexpression under laser irradiation (660 nm, 20 mW cm^−2^, 5 min). In contrast, both FCCPAO NAs and FSSPAO NAs with robust oxygen‐carrying capacity effectively attenuated HIF‐1α expression under the same conditions, demonstrating the vital role of oxygen supply in mitigating tumor cell hypoxia (Figure 4H; Figure S18, Supporting Information). Additionally, while ATO could effectively reduce cellular oxygen consumption, it had minimal impact on intracellular HIF‐1α expression in both 4T1 and CT26 cells under hypoxia (Figure 4H; Figure S18, Supporting Information), which is consistent with previous findings.^[^ ^25^ ^]^ Taken together, these results underscored that PDT modality significantly increased cellular HIF‐1α expression, while sufficient oxygen supply played a pivotal role in combatting this process. Meanwhile, ATO had marginal impact on HIF‐1α expression.

### 2.10 In Vitro PDT Efficacy

Efficient in vitro cellular uptake and hypoxia alleviation of FSSPAO NAs have spurred us to further investigate their potential for enhancing PDT efficacy. Such an oxygen nano‐tank with high PPa loading and closed‐loop hypoxia alleviation capacity would offer the prospect of inducing substantial bursts of ROS under laser irradiation, even under hypoxic conditions. We first assessed the ROS generation in 4T1 and CT26 cells exposed to various formulations utilizing the DCFH‐DA probe. As depicted in Figures 4I and S19 (Supporting Information), the fluorescence signals emitted by 4T1 and CT26 cells treated with PPa Sol were notably more pronounced under normoxic conditions than under hypoxia, suggesting the significant impact of hypoxia on cellular ROS production and PDT efficacy. Remarkably, FSSPAO NAs generated much more ROS than oxygen‐free NAs (HSSPAO NAs) and ATO‐free NAs (FSSPO NAs and FCCPAO NAs) under hypoxic conditions (Figure 4I; Figure S19, Supporting Information). The excellent ROS generation capability of FSSPAO NAs can be attributed to the favorable PPa/ATO nanoassembly, excellent oxygen‐carrying ability, good stability, efficient cellular uptake, effective ACQ relief, and closed‐loop hypoxia alleviation.

Subsequently, we evaluated the in vitro cytotoxicity of FSSPA NAs against normal cells (L929 cells) and tumor cells (4T1 and CT26 cells) using MTT assay. As shown in Figure S20A (Supporting Information), FSSPA NAs exhibited negligible effects on L929 cells, suggesting favorable biocompatibility of FSSPA NAs. As depicted in Figures S20B and S21A (Supporting Information), PPa Sol and ATO Sol exhibited minimal dark toxicity under both normoxic and hypoxic conditions. Under laser irradiation, PPa Sol, HSSPA NAs, FCCPA NAs and FSSPA NAs elicited higher cytotoxicity under normoxic conditions than that under hypoxic conditions, suggesting the detrimental impact of hypoxia on PDT (Figure 4J; Figure S21B, Supporting Information). Of particular significance, there was negligible difference in cytotoxicity between FSSP NAs/L and FSSPA NAs/L under normoxic conditions, while FSSPAO NAs/L demonstrated superior cytotoxic than that of FSSPO NAs/L under hypoxic conditions (Figure 4K; Figure S21C and D, Supporting Information). These findings indicated that ATO alone is insufficient in significantly enhancing the photodynamic cytotoxicity of FSSP in the absence of oxygen supply, while ATO‐inhibited oxygen consumption substantially potentiated the cytotoxicity of FSSPAO NAs under hypoxic conditions. Significantly, under hypoxic conditions, FSSPAO NAs displayed very similar photodynamic cytotoxicity when compared to FSSP NAs under normoxic conditions (Figure 4K; Figure S21C and D, Supporting Information), suggesting that FSSPAO NAs with the ability of oxygen supply and oxygen consumption inhibition effectively improved PDT efficiency against hypoxic tumors. Moreover, FSSPAO NAs elicited more potent photodynamic cytotoxicity under hypoxic conditions compared to that of PPa Sol, HSSPAO NAs and FCCPAO NAs (Figure 4L; Figure S21E, Supporting Information), which should be ascribed to its favorable colloidal stability (Figure 3D), efficient cellular uptake (Figure 4A,B), sufficient oxygen supply (Figure 3H) and alleviative ACQ effect (Figure 3K).

### 2.11 Pharmacokinetics and Biodistribution

**Figure 5:**
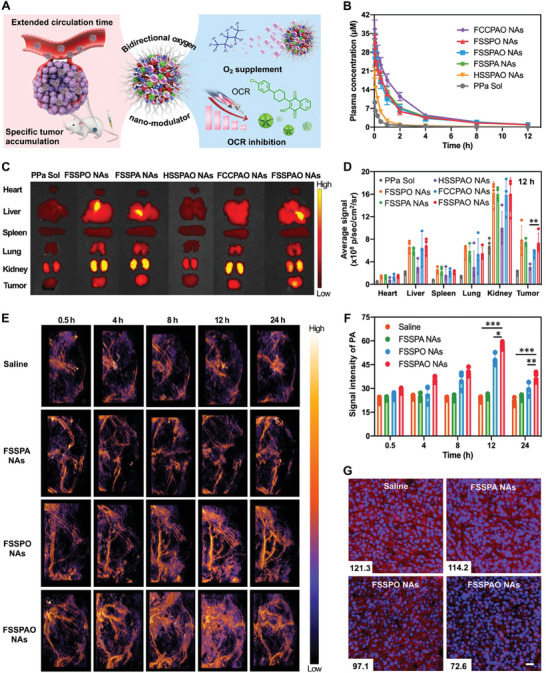
In vivo pharmacokinetics, biodistribution and relief of tumor hypoxia in vivo. A) Schematic representation of in vivo delivery disposition and tumor hypoxia relief by the oxygen nano‐tank. B) The concentration‐time curves of PPa Sol, HSSPAO NAs, FSSPA NAs, FSSPAO NAs, FSSPO NAs and FCCPAO NAs after a single intravenous administration at a PPa equivalent dose of 2 mg kg^−1^ (n = 5). C) Fluorescent imaging of organs and tumors in 4T1 tumor‐bearing BALB/c mice at 12 h post administration. D) Quantitative results of fluorescence intensity in the major organs and tumors of 4T1 tumor‐bearing BALB/c mice at 12 h (n = 4). Data were analyzed by IVIS spectrum small‐animal in vivo imaging system. E) In vivo intratumoral photoacoustic images of oxyhemoglobin (λ = 1064 nm) in 4T1 tumor‐bearing BALB/c mice after intravenous injection at different time intervals (0.5, 4, 8, 12 and 24 h). F) Quantitative results of the photoacoustic intensity in tumors at different time intervals (0.5, 4, 8, 12 and 24 h) after different treatments (n = 4). Data were analyzed by 3D Slicer image computing platform. G) Representative immunofluorescence images and quantitative analysis of tumor slices stained by Hypoxyprobe‐1 (pseudo‐red color). The cell nucleus was stained with DAPI (Scale bar: 20 µm).

In vivo pharmacokinetics and biodistribution of nanomedicines play crucial roles in determining their ultimate therapeutic efficacy and safety. PEGylation modification and fluorination‐stabilized nanoassembly would significantly extend the circulation time of fluorinated NAs in the blood stream following intravenous administration (**Figure** **5**A). The pharmacokinetic profiles of PPa Sol, FSSPO NAs, FSSPA NAs, HSSPAO NAs, FCCPAO NAs and FSSPAO NAs were investigated in SD rats at the same molar dose of PPa (2 mg kg^−1^). As shown in Figure 5B and Table S6 (Supporting Information), PPa Sol was rapidly cleared from the blood due to its short biological half‐life. Moreover, the non‐fluorinated HSSPAO NAs, characterized by inferior stability, also exhibited poor pharmacokinetic behavior (Figure 5B). As expected, the fluorinated NAs (FSSPO NAs, FSSPA NAs, FCCPAO NAs and FSSPAO NAs) significantly extended the blood circulation time of PPa (Figure 5B). As shown in Figure 5B, FCCPAO NAs exhibited a slight advantage over FSSPAO NAs, which should be attributed to better chemical stability of the non‐sensitive FCCP conjugate. Notably, no substantial difference was observed in the pharmacokinetic profiles of FSSPO NAs, FSSPA NAs and FSSPAO NAs (Figure 5B), indicating that neither coaasembly with ATO nor oxygencarry had a significant impact on the in vivo delivery fate of FSSP. Taken together, these results indicated that fluorination modification and molecular nanoassembly collaboratively served to improve the bioavailability of PPa with remarkably increased area under the plasma concentration‐time curve (AUC, Table S6, Supporting Information). Favorable pharmacokinetic properties would undoubtedly enhance the tumor‐specific accumulation of nanomedicines through the enhanced permeability and retention (EPR) effect.

FSSPAO NAs were not only able to perform as an oxygen nano‐tank for closed‐loop hypoxia alleviation, but also to serve as a self‐indicating nanomedicine for real‐time tracing and imaging by using the fluorescence of PPa. Particularly, it is very necessary to figure out an optimal time window with high drug accumulation for conducting laser irradiation in anticancer PDT. In this section, the *ex vivo* biodistribution of PPa Sol, FSSPO NAs, FSSPA NAs, HSSPAO NAs, FCCPAO NAs and FSSPAO NAs was explored in 4T1 tumor‐bearing BALB/c mice. As depicted in Figure S22 (Supporting Information), these formulations exhibited comparable *ex vivo* biodistribution at an early time point (4 h). Remarkably, a gradual increase trend was observed in tumor fluorescence intensity of the fluorinated NAs (FSSPA NAs, FCCPAO NAs and FSSPAO NAs) from 4 h to 12 h (Figure 5C,D; Figure S22, Supporting Information). By contrast, PPa Sol and HSSPAO NAs displayed a decreasing trend under the same conditions (Figure 5C,D; Figure S22, Supporting Information), which could be attributed to their poor pharmacokinetics (Figure 5B). Notably, despite the better pharmacokinetic performance of FCCPAO NAs, FSSPAO NAs demonstrated even higher fluorescence intensity in tumors than that of FCCPAO NAs under the same conditions, especially at 12 h (Figure 5C,D; Figure S22, Supporting Information). The relatively weaker fluorescence intensity of FCCPAO NAs could be attributed to the ACQ dilemma of PPa trapped in the non‐sensitive nanostructure (Figure S10, Supporting Information). Additionally, negligible difference in intratumoral fluorescence intensity was observed among FSSPO NAs, FSSPAO NAs and FSSPA NAs (Figure 5C,D; Figure S22, Supporting Information), which was consistent with the pharmacokinetic results (Figure 5B). These results suggested that FSSPAO NAs not only substantially prolonged the blood circulation time but also facilitated tumor‐specific accumulation of PPa, which would certainly benefit anticancer PDT.

### 2.12 Bidirectional Oxygen Modulation‐Facilitated Hypoxia Alleviation In Vivo

It was expected that such an oxygen nano‐tank with favorable pharmacokinetic behavior and tumor accumulation would create a time window with elevated tumor oxygen levels through closed‐loop hypoxia alleviation (Figure 5A). To evaluate the efficacy of hypoxia relief, we employed a photoacoustic imaging system to detect oxyhemoglobin signals within 4T1 tumor‐bearing BALB/c mice treated with saline, FSSPA NAs, FSSPO NAs and FSSPAO NAs. As shown in Figure 5E,F, the intratumoral photoacoustic signals of mice receiving FSSPO NAs and FSSPAO NAs were gradually increased from 0.5 h to 12 h (Figure 5C,D; Figure S22, Supporting Information). By contrast, there was no obvious variation of intratumoral photoacoustic signals in the mice treated with saline and FSSPA NAs from 0.5 to 24 h (Figure 5E,F). These results suggested that oxygen‐carrying NAs significantly improved the intratumoral oxygen content in a time‐dependent manner, while ATO‐containing FSSPA NAs without oxygen loading didn't yield a significant elevation of intratumoral oxygen levels. That is, oxygen consumption inhibition alone plays a very limited role in hypoxia relief, due to the inherently low oxygen levels in solid tumors. Significantly, the nano‐tank (FSSPAO NAs) with oxygen supply and consumption inhibition capacity demonstrated much stronger photoacoustic signals in tumors than any other formulations under the same conditions. Moreover, the peak oxygen content in tumor treated with FSSPAO NAs was at 12 h (Figure 5E,F), which showed a very similar trend with the tumor accumulation results (Figure 5C,D).

Additionally, Hypoxyprobe‐1 (pimonidazole hydrochloride) was also used to further detect tumor hypoxia after different treatments. As exhibited in Figure 5G, the tumor slices of mice receiving saline and FSSPA NAs showed large areas of hypoxia. Moreover, FSSPO NAs groups with oxygen supplement capability exhibited a partial mitigation of hypoxic conditions in tumors (Figure 5G). As expected, the tumor slices of mice receiving FSSPAO NAs exhibited the smallest hypoxic area, which was consistent with the photoacoustic imaging results (Figure 5E,F). Taken together, we developed an oxygen supply/consumption inhibition bifunctional nanomedicine to create a time window with elevated tumor oxygen levels, which showed distinct advantages in terms of oxygen modulation in vitro and in vivo. Such a credible oxygen‐modulating nanomedicine would certainly boost PPa‐mediated PDT against solid tumors.

### 2.13 Hypoxia Alleviation‐Enabled Photodynamic Tumor Eradication

**Figure 6:**
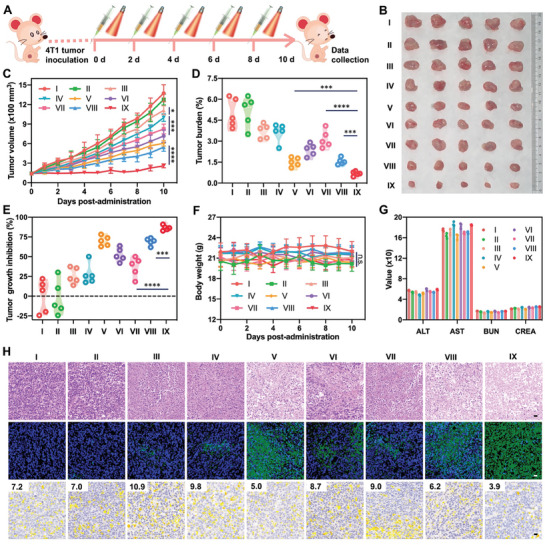
In vivo antitumor activity of the oxygen nano‐tank against 4T1 xenograft breast tumors (n = 5). A) Schematic representation of experimental design. B) Images of tumors after the last treatment. C) Tumor growth profiles treated with different formulations. D) Tumor burden after the last treatment. E) Tumor growth inhibitory rate after the last treatment. F) Body weight changes of BALB/c mice bearing 4T1 xenograft breast tumors during treatment with different formulations. G) Hepatorenal function evaluation after the last treatment (AST: aspartate aminotransferase; ALT: alanine aminotransferase, BUN: blood urea nitrogen, CREA: creatinine). H) H&E staining, TUNEL assay, HIF‐1α immunohistochemical staining and H‐SCORE analysis results of tumor sections after the last treatment (Scale bar: 20 µm). The I, II, III, IV, V, VI, VII, VIII and IX represent saline, ATO Sol, PPa Sol/L, ATO/PPa mixture/L, FSSPO NAs/L, FSSPA NAs/L, HSSPAO NAs/L, FCCPAO NAs/L and FSSPAO NAs/L, respectively. The “L” represents laser irradiation (660 nm, 20 mW cm^−2^, 5 min).

**Figure 7:**
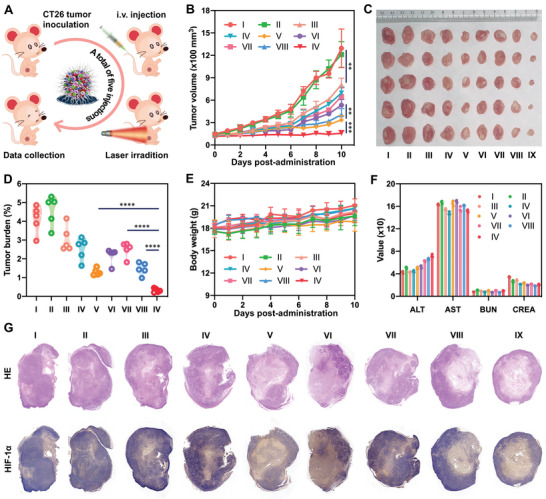
In vivo antitumor activity of the nano‐tank against CT26 xenograft colon adenocarcinoma (n = 5). A) Schematic representation of experimental design. B) Tumor growth profiles treated with different formulations. C) Images of tumors after the last treatment. D) Tumor burden after the last treatment. E) Body weight changes of BALB/c mice bearing CT26 xenograft tumors during treatment with different formulations. F) Hepatorenal function evaluation after the last treatment (AST: aspartate aminotransferase; ALT: alanine aminotransferase, BUN: blood urea nitrogen, CREA: creatinine). G) H&E and immunohistochemical staining results of CT26 tumor sections after the last treatment. The I, II, III, IV, V, VI, VII, VIII and IX represent saline, ATO Sol, PPa Sol/L, ATO/PPa mixture/L, FSSPO NAs/L, FSSPA NAs/L, HSSPAO NAs/L, FCCPAO NAs/L and FSSPAO NAs/L, respectively. The “L” represents laser irradiation (660 nm, 20 mW cm^−2^, 5 min).

The favorable nanoassembly feature, oxygen carrying capacity, prodrug activation/drug release patterns, photodynamic efficiency, pharmacokinetics, tumor‐specific accumulation, as well as hypoxia alleviation of FSSPAO NAs made it as a highly promising photodynamic nanomedicine for cancer therapy. In this section, we explored the antitumor efficiency and safety in 4T1 breast and CT26 colon tumor‐bearing mouse models (**Figures** **6**A and **7**A), respectively. In brief, saline, ATO Sol, PPa Sol, PPa/ATO mixture, FSSPO NAs, FSSPA NAs, HSSPAO NAs, FCCPAO NAs and FSSPAO NAs were intravenously administrated to 4T1 breast and CT26 colon tumor‐bearing mice for a total of five injections, respectively. According to the biodistribution findings (Figure 5C,D; Figure S22, Supporting Information), PPa Sol, PPa/ATO mixture and HSSPAO NAs‐treated groups underwent laser exposure (660 nm, 20 W cm^−2^) for 5 min at 4 h post‐administration, respectively. Meanwhile, FSSPO NAs, FSSPA NAs, FCCPAO NAs and FSSPAO NAs‐treated groups were exposed to laser (660 nm, 20 W cm^−2^) for 5 min at 12 h post administration, respectively.

As depicted in Figures 6B–E and 7B–D, ATO Sol, PPa Sol/L and ATO/PPa mixture/L had unsatisfactory outcomes, due to the inferior pharmacokinetics and tumor‐specific accumulation of free drug solutions. Oxygen‐carrying FSSPO NAs/L and ATO‐containing FSSPA NAs/L displayed modest antitumor activities, indicating the limitations of either oxygen supply or consumption inhibition strategy in addressing tumor hypoxia challenge. Moreover, the non‐sensitive FCCPAO NAs/L also elicited a moderate therapeutic effect, which could be attributed to the ACQ (Figure 3K) and ATO release dilemma (Figure 3O). As expected, the oxygen nano‐tank (FSSPAO NAs) demonstrated distinct advantages over other formulations under the same conditions, effectively suppressing tumor progression in both 4T1 breast and CT26 colon tumor models (Figures 6B–E and 7B–D). The potent antitumor efficacy of FSSPAO NAs should be ascribed to its multiple advantages throughout drug delivery process, including favorable colloidal stability (Figure 3D), ACQ mitigation (Figure 3K), on‐demand drug release (Figure 3M–O), efficient cellular uptake (Figure 4A,B; Figures S13 and S14, Supporting Information), closed‐loop hypoxia alleviation (Figure 4C–H), high‐yield ROS generation (Figure 4I), favorable pharmacokinetics (Figure 5B) and site‐specific accumulation (Figure 5C,D; Figure S22, Supporting Information). Similar results were found in the H&E staining results, with widespread apoptosis and necrosis in tumor sections (Figures 6H and 7G). Moreover, HIF‐1α expression in tumor tissues was also investigated after treatments. As shown in Figures 6H and 7G, ATO alone had almost no influence on HIF‐1α expression, while the intratumoral HIF‐1α levels were significantly increased after treatment with PPa‐containing formulations (PPa Sol/L, ATO/PPa mixture/L, FSSPA NAs/L and HSSPAO NAs/L) under laser irradiation, indicating PDT‐aggravated tumor hypoxia. Notably, both FSSPO NAs and FSSPAO NAs effectively attenuated HIF‐1α expression, demonstrating the essential contribution of oxygen supply to hypoxia relief (Figures 6H and 7G). There was no significant abnormality seen in body weight and hematological parameters of both 4T1 breast and CT26 colon tumor‐bearing mice during treatments (Figures 6F,G and 7E,F). Moreover, all these formulations did not cause hemolysis, revealing good biocompatibility for intravenous administration (Figure S23, Supporting Information). Meanwhile, no noticeable histological variation was observed in heart, liver, spleen, lung and kidney (Figures S24 and S25, Supporting Information). These results indicated that FSSPAO NAs not only performed potent antitumor activity, but also didn't cause systemic toxicity throughout treatment process.

## 3 Conclusion

The development and clinical translation of oxygen‐carrying nanomedicines for the mitigation of tumor hypoxia are still impeded by inferior biocompatibility of nanocarrier materials and unsatisfactory kinetics in loading and releasing oxygen. Particularly, it would be futile if nanocarrier materials can't be developed into pharmaceutical excipients. Translation of new carrier materials into excipients is full of challenges, especially for intravenous administration. Fortunately, we found that a small‐molecule fluorination prodrug nanoassembly showed striking ability to carry oxygen, even much better than the carrier‐based oxygen nanocarrier. It's worth noting that the prodrug NAs were fabricated without the help of any nanocarrier materials. Instead, only a small amount of DSPE‐PEG_2k_ was employed as modifier to endow the NAs with better colloidal stability and drug delivery performance. Notably, DSPE‐PEG_2k_, as a well‐established pharmaceutical excipient, has been approved for preparation of long‐circulating nanomedicines. Moreover, we found that exogenous oxygen supply by fluorination prodrug nanoassembly alone was insufficient to efficiently address the challenge of tumor hypoxia. More than that, we detected a rapid surge in cellular OCR upon the exogenous oxygen supplementation to hypoxic cancer cells. These findings underscored the equal importance of inhibiting oxygen consumption alongside oxygen supplementation in the endeavor to effectively alleviate tumor hypoxia.

In this study, we successfully developed a carrier‐free oxygen nano‐tank in basis of modular prodrug design and small‐molecule prodrug/drug nanoassembly technique. Significantly, such a uniquely engineered nanomedicine was able to create a hypoxia alleviation window enabling potent PDT against two solid tumor models by integrating fluoridized prodrug‐mediated oxygen supply and ATO‐blocked oxygen consumption inhibition into a single nanosystem. We described and validated the feasibility of a small‐molecule fluoridized prodrug‐engineered nanoassembly for efficient and stable oxygen loading and delivery. More important, the precise co‐assembly with ATO not only preserved the oxygen‐carrying efficiency of the nanoassembly but also enhanced its oxygen enrichment capacity. The nano‐tank (FSSPAO NAs) exhibited many advantages, including facile fabrication, high drug co‐loading and oxygen‐carrying efficiency, long circulation time in the blood, favorable tumor‐specific accumulation, on‐demand prodrug activation and ATO release. These advantages collectively led to effective oxygen enrichment in solid tumors, significantly potentiating PPa‐mediated PDT with good safety under laser irradiation in vitro and in vivo. Such a modular fluorination prodrug‐based oxygen‐enriching strategy holds promise for amplifying the efficacy of other hypoxia‐hampered anticancer therapies in clinic, such as chemotherapy, radiotherapy, gene therapy, as well as immunotherapy.

## Conflict of Interest

The authors declare no conflict of interest.

## Supporting information

Supporting Information

## Data Availability

Research data are not shared.
