# Supplementary material for: Modular Prodrug‐Engineered Oxygen Nano‐Tank With Outstanding Nanoassembly Performance, High Oxygen Loading, and Closed‐Loop Tumor Hypoxia Relief
Source: Adv Sci (Weinh). 2024 Jul 10;11(34):2405583. doi: 10.1002/advs.202405583 (PMC11425644; doi:10.1002/advs.202405583)
Supplement: Supplementary file 1 — Supporting Information [file ADVS-11-2405583-s001.docx]

**Supporting Information**

**Modular Prodrug-engineered Oxygen Nano-tank With Outstanding Nanoassembly Performance, High Oxygen Loading, and Closed-loop Tumor Hypoxia Relief**

Fujun Yang^1,2^, Shumeng Li^3^, Qingyu Ji^1,2^, Hongyuan Zhang^1,2^, Mingyang Zhou^4^, Yuequan Wang^1,2^, Shenwu Zhang^1,2^, Jin Sun^1,2^, Zhonggui He^1,2^, Cong Luo^1,2*^

^1^Department of Pharmaceutics, Wuya College of Innovation, Shenyang Pharmaceutical University, Shenyang 110016, PR China

^2^Joint International Research Laboratory of Intelligent Drug Delivery Systems, Ministry of Education, Shenyang Pharmaceutical University, Shenyang 110016, PR China

^3^Department of Pharmaceutical Analysis, School of Pharmacy, Shenyang Pharmaceutical University, Shenyang 110016, PR China

^4^Department of Chemistry, University of Pennsylvania, Philadelphia, Pennsylvania 19104-6323, United States

E-mail: luocong@syphu.edu.cn

**Supplemental experimental methods**

**Chemicals and reagents.** Pyropheophorbide a (PPa) was sourced from Shanghai Dibai Chemical Technology Co. Ltd, China. Atovaquone (ATO), hexylamine and 1H,1H-perfluorohexylamine were supplied by Aladdin Biochemical Technology Co. Ltd. (Shanghai, China). Dithiodiglycolic acid and adipic acid were bought from Macklin Biochemical Co., Ltd (Shanghai, China). Dithiothreitol (DTT), 4-dimethylaminopyrideine (DMAP), 1-hydroxybenzotriazole (HOBt) and 1-(3-Dimethylaminopropyl)-3-ethylcarbodiimide hydrochloride (EDCI) were purchased from TCI (Shanghai) Development Co., Ltd (Shanghai, China). 1,2-distearoylsn-glycero-3-phosphoethanolamine-N-[methoxy(polyethyleneglycol)-2000 (DSPE-PEG_2k_) was supplied by AVT Shanghai Pharmaceutical Technology Co., Ltd. Mitochondrial Tracker Green, singlet oxygen sensor green (SOSG) assay kit, (2,7-dichlorodihydrofluorescein diacetate (DCFH-DA) assay kit, the RPMI 1640 cell culture medium, penicillin-streptomycin and 3-(4,5-dimthyl-2-thiazolyl)-2,5-dipphenyl-2H-terazolium bromide (MTT) were provided by Dalian Meilun Biotechnology Co. Ltd., China. Mitochondria complex III testing kit was purchased from Beijing Solarbio Science & Technology Co., Ltd. (Beijing, China). Anti-HIF-1α monoclonal antibody was obtained from Abcam Inc., USA. Cell culture plates were obtained from NEST Biotechnology Co., Ltd (Wuxi, China). Other solvents and chemicals were of analytical grade and used without further purification.

**Synthesis of fluoridized prodrug (FSSP)**. PPa (1 mmol) was dissolved in dichloromethane and the solution of EDCI (2 mmol), HOBt (2 mmol) and DMAP (0.4 mmol) was dripped into the system in ice bath for 2 h. After that, ethylene glycol (2 mmol) was added into the reaction solution for 48 h under the protection of nitrogen at room temperature. Then, an important chemical intermediate of the photosensitizer with hydroxyl group (PPa-EG) was separated out by preparative liquid chromatography (100% methanol as mobile phase). Dithiodiglycolic acid (1 mmol) was dissolved in acetic anhydride to react for 2 h with nitrogen atmosphere protection at room temperature, then, methylbenzene was poured into the reaction solution followed by evaporating under vacuum for several times. Subsequently, anhydrous dichloromethane was utilized to dissolve the crude product, and then DMAP (0.1 mmol) and PPa-EG (0.5 mmol) were added to react for 24 h at room temperature. After that, the product (1 mmol) obtained above, EDCI (2 mmol), HOBt (2 mmol) were dissolved in anhydrous dichloromethane, then 1H,1H-perfluorohexylamine (1 mmol) was dripped into the reaction solution under stirring for 48 h under nitrogen at room temperature. Finally, preparative liquid chromatography (acetonitrile/water (95:5) as the mobile phase) was applied to obtain the target fluoridized prodrug (FSSP). Mass spectrometry and ^1^H NMR were leveraged for certifying the structure of FSSP, respectively.

**Synthesis of control prodrugs (FCCP and HSSP)**. Both FCCP and HSSP were synthesized by a similar synthesis route with FSSP except that dithiodiglycolic acid was substituted with adipic acid, and 1H,1H-perfluorohexylamine was replaced by hexylamine, respectively. Mass spectrometry and ^1^H NMR were also utilized to characterize the successful synthesis of these PPa prodrugs.

**Ultraviolet and fluorescence spectra.** The ultraviolet and fluorescence spectra of PPa, FSSP, FCCP and HSSP solution were characterized by multimode microplate reader (Thermo Scientific, USA) at a PPa equivalent concentration of 5 μg mL^-1^. The ultraviolet spectra were measured between 300 and 800 nm. The fluorescence spectra were obtained at excitation of 415 nm and at emission of 675 nm, respectively.

**Preparation and characterization of self-assemblies.** The non-PEGylated self-assemblies of PPa prodrugs (NFSSP NAs, NFCCP NAs and NHSSP NAs) were conducted by one-step nanoprecipitation method. In brief, 5.12 mg of FSSP (or 4.94 mg of FCCP or 4.13 mg of HSSP) were dissolved in a 0.5 mL of ethanol/tetrahydrofuran mixture (1:1, v/v), then, the mixed solution was added dropwise into deionized water (5 mL) under stirring (1200 rpm). Afterwards, the organic solvents were removed by vacuum-rotary evaporation at 30 °C. Moreover, PEGylated self-assemblies (FSSP NAs, FCCP NAs and HSSP NAs) were fabricated by a similar procedure except for adding DSPE-PEG_2k_ (20%, w/w) into the mixtures before dropping into deionized water. In addition, PEGylated PPa self-assemblies (PPa NAs) was also conducted by one-step nanoprecipitation method. In brief, 5.34 mg of PPa were dissolved in a 0.5 mL of ethanol/tetrahydrofuran mixture (1:1, v/v), then, the mixed solution was added dropwise into deionized water (5 mL) under stirring (1200 rpm). Afterwards, the organic solvents were removed by vacuum-rotary evaporation at 30°C. Finally, the hydrodynamic diameters and Zeta potentials of PPa NAs, FSSP NAs, FCCP NAs and HSSP NAs were determined by Zetasizer (NanoZS, Malvern Co., UK), and the calculated LogP (CLogP) values of FSSP and HSSP were calculated by MarvinSketch.

The loading rate was calculated by the following equations: DL_PPa of FSSP NAs_ = 0.8 × M_PPa_ / M_FSSP NAs_ × 100%; DL_1H,1H-perfluorohexylamine of FSSP NAs_ = 0.8 × M_1H,1H-perfluorohexylamine_ / M_FSSP NAs_ × 100%. M_PPa_ is the molecular weight of PPa, M_1H,1H-perfluorohexylamine_ is the molecular weight of 1H,1H-perfluorohexylamine, respectively.

**Oxygen-carrying capacity of fluoridized self-assemblies**. The prodrug-loaded PLGA NPs (FSSP-PLGA NPs, FCCP-PLGA NPs and HSSP-PLGA NPs) were fabricated via emulsion solvent evaporation technique. Briefly, 5.12 mg of FSSP (or 4.94 mg of FCCP or 4.13 mg of HSSP) and 60 mg of PLGA were dissolved in 4 mL of ethanol/tetrahydrofuran mixture (1:1, v/v). After that, the mixed solution was added into deionized water (2 mL) containing 1% PVA, followed by sonication for 10 min at 0 °C. The organic solvent was also removed through vacuum-rotary evaporation at room temperature. Subsequently, the obtained suspensions were centrifuged for 30 min and then dispersed in deionized water (5 mL). Furthermore, oxygen-saturated FSSPO NAs, FCCPO NAs, HSSPO NAs, FSSPO-PLGA NPs, FCCPO-PLGA NPs and HSSPO-PLGA NPs were obtained by letting pure O_2_ in with a constant flow rate (5 L/min) for 30 min. The loading rate was calculated by the following equations: DL_1H,1H-perfluorohexylamine of FSSP-PLGA NPs_ = W_FSSP_ × (M_1H,1H-perfluorohexylamine_ / M_FSSP_) / (W_FSSP_ + W_PLGA_) × 100%. M_1H,1H-perfluorohexylamine_ and M_FSSP_ is the molecular weight of 1H,1H-perfluorohexylamine and FSSP, respectively. W_FSSP_ and W_PLGA_ is the weight of FSSP and PLGA, respectively.

The dissolved oxygen meter (WLDO-300, Shanghai Water Inst Technology CO. Ltd.) was employed to assess oxygen-carrying capacity. Briefly, 5 mL of PBS (pH 7.4), FSSPO NAs, FCCPO NAs, HSSPO NAs, FSSPO-PLGA NPs, FCCPO-PLGA NPs and HSSPO-PLGA NPs (equal to 2 μmol mL^-1^ of 1H,1H-perfluorohexylamine or hexylamine) were added into 15 mL of deoxygenated water. According to preclinical setup of sonication, an external sonication with a 30% amplitude (constant frequency of 30 kHz) was applied to trigger oxygen release. The oxygen concentration was further recorded via dissolved oxygen meter (WLDO-300, Shanghai Water Inst Technology CO. Ltd.). The oxygen loading capability of 1 mL of NAs (equal to 2 μmol mL^-1^ of 1H,1H-perfluorohexylamine or hexylamine) was calculated according to the following formula: Oxygen loading capacity = increased oxygen concentration after the addition of NAs × total volume of final solution/volume of NAs.

**Real-time cellular oxygen consumption rates**. The real-time OCR of 4T1 and CT26 cells under normoxic and hypoxic conditions were real-time monitored using a BBoxiProbe™ R01 kit according to the manufacturer's protocol. Briefly, 4T1 or CT26 cells were seeded in 96-well plates with transparent bottoms and black sides at a density of 5 × 10^3^ cells/well under normoxic and hypoxic conditions, respectively. After overnight incubation, the 4T1 or CT26 cells were further cultured with PBS (pH 7.4) and FSSPO NAs (equal to 10 μM of 1H,1H-perfluorohexylamine), respectively. After that, 10 μL of oxygen fluorescent probe and 100 μL of blocking buffer were immediately added. Finally, the fluorescence of the plates was recorded on a multimode microplate reader (Thermo Scientific, USA) over 60 min at 5-min intervals. The cellular oxygen consumption rates were calculated using the following formula:

OCR (%) = (the final fluorescence in PBS or FSSPO NAs-treated cells under normoxic or hypoxic conditions − initial fluorescence in PBS or FSSPO NAs-treated cells under normoxic or hypoxic conditions)/(final fluorescence in PBS-treated cells under normoxic conditions − initial fluorescence in PBS-treated cells under normoxic conditions) × 100%.

**Preparation and characterization of binary NAs.** In order to enable closed-loop hypoxia alleviation and photodynamic tumor eradication, a bidirectional oxygen nano-tank self-assembled by FSSP and ATO was fabricated by nano-precipitation method. Briefly, 5.12 mg of FSSP and 1.84 mg of ATO were dissolved in a 0.5 mL of ethanol/tetrahydrofuran mixture (1:1, v/v), respectively. To optimize the nanoassembly engineering process conditions, the mixtures of FSSP and ATO at various molar ratios of 4:1, 3:1, 2:1, 1:1, 1:2, 1:3 and 1:4 (FSSP/ATO) were obtained by mixing above solutions. In addition, DSPE-PEG_2k_ (20 wt%) was further added into above mixture. Then, deionized water was added dropwise into of the mixed solutions under robust stirring to prepare a series of PEGylated FSSP/ATO hybrid NAs (FSSPA NAs). After that, the organic solvents in nanosystems were removed through vacuum-rotary evaporation. To confirm the successful co-assembly of FSSP and ATO, elemental mapping was performed at the nanostructural level by scanning electron microscopy (SEM) with energy dispersive X-ray spectrometry (EDS). Moreover, the hydrodynamic diameters and morphologies of FSSPA NAs were determined by Zetasizer (NanoZS, Malvern Co., UK) and transmission electron microscopy (TEM) (JEOL 100CX II, Japan), respectively.

After determining that molar ratio of 2:1 (FSSP/ATO) was the optimal nanoassembly formulation, the PEGylated FCCPA NAs and HSSPA NAs at the same molar ratio of 2:1 (prodrug/ATO) were further constructed by a similar route with FSSPA NAs except that FSSP was replaced by FCCP and HSSP, respectively. Similarly, the hydrodynamic diameters and the morphologies of FCCPA NAs and HSSPA NAs were also determined by Zetasizer (NanoZS, Malvern Co., UK) and transmission electron microscopy (TEM) (JEOL 100CX II, Japan), respectively. The loading rate of ATO in FSSPA NAs was calculated by the following equations: DL_ATO of FSSPA NAs_ = 0.8 × (a×M_ATO_) / (a × M_ATO_ + b × M_FSSP_) × 100%. M_ATO_ is the molecular weight of ATO, M_FSSP_ is the molecular weight of FSSP, and a and b are the molar ratios of ATO and FSSP, respectively.

**Colloidal stability.** To study the colloidal stability of FSSPA NAs, FCCPA NAs and HSSPA NAs under simulated physiological conditions, nanoassemblies (equal to 1 μmol mL^-1^ of PPa) were incubated in PBS (pH 7.4) supplemented with 10% FBS in a shaker box at 37 °C. At presupposed time intervals (0, 2, 4, 6, 8, 10 and 12 h), the particle sizes of FSSPA NAs, FCCP NAs and HSSPA NAs were measured by Zetasizer (Nano ZS, Malvern Co., UK). In addition, the colloidal stability of FSSPA NAs in solutions of different pH was further determined. Briefly, FSSPA NAs (equal to 1 μmol mL^-1^ of PPa) were incubated in PBS (pH 5.6), PBS (pH 6.8) and PBS (pH 7.4) in a shaker box at 37 °C, respectively. At presupposed time intervals (0, 2, 4, 6, 8, 10 and 12 h), the particle sizes of FSSPA NAs were measured by Zetasizer (Nano ZS, Malvern Co., UK). In order to investigate the stability of FSSPA NAs under laser irradiation, FSSPA NAs (equal to 1 μmol mL^-1^ of PPa) were irradiated with 660 nm laser (20 mW cm^-2^) for 5 min. After that, the particle sizes of FSSPA NAs were measured by Zetasizer (Nano ZS, Malvern Co., UK) at presupposed time intervals (0, 2, 4, 6, 8, 10 and 12 h). In addition, the colloidal stability of FSSPA NAs in PBS (pH 7.4) was further determined. Briefly, FSSPA NAs (equal to 1 μmol mL^-1^ of PPa) were incubated in PBS (pH 7.4) in a shaker box at 37 °C. At presupposed time intervals (0, 12, 24, 36, 48 and 72 h), the particle sizes of FSSPA NAs were measured by Zetasizer (Nano ZS, Malvern Co., UK).

**Molecular co-assembly mechanisms.** The co-assembly mechanisms of FSSP and ATO were investigated by molecular docking simulation method. The 3-dimensional structures of FSSP and ATO were obtained using the Autodock Vina software. To further verify the intermolecular forces, intermolecular interaction breakers including SDS and urea (200 mM) were utilized to co-incubated with FSSPA NAs, FCCPA NAs and HSSPA NAs, respectively. The particle size changes of nanoassemblies were measured by Zetasizer (NanoZS, Malvern Co., UK). Furthermore, the ultraviolet spectra of PPa Sol, FSSPA NAs, FCCPA NAs and HSSPA NAs were analyzed by multimode microplate reader (Thermo Scientific, USA) to examine the existence of π-π stacking interaction.

**Oxygen-carrying capacity of binary NAs**. Oxygen-saturated FSSPAO NAs, FCCPAO NAs and HSSPAO NAs were obtained by letting pure O_2_ in with a constant flow rate (5 L/min) for 30 min. Then, 5 mL of PBS (pH 7.4), FSSPAO NAs, FCCPAO NAs, HSSPAO NAs (equal to 2 μmol mL^-1^ of 1H,1H-perfluorohexylamine or hexylamine) were added into 15 mL of deoxygenated water. Afterwards, an external sonication was applied to trigger oxygen release, and oxygen concentration was further recorded via dissolved oxygen meter (WLDO-300, Shanghai Water Inst Technology CO. Ltd.). The oxygen loading capability of 1 mL of NAs (equal to 2 μmol mL^-1^ of 1H,1H-perfluorohexylamine or hexylamine) was calculated according to the following formula:

Oxygen loading capacity = increased oxygen concentration after the addition of NAs × total volume of final solution/volume of NAs.

**Redox-responsive prodrug activation and ACQ relief**. To investigate the redox-responsive activation profile of prodrugs, 0.5 μmol FSSP or FCCP were incubated in 30 mL release medium containing 1 mM of DTT at 37 °C, respectively. At the preset time points, the typical peaks of PPa-EG and FSSP were analyzed using HPLC. Chromatographic separation was carried out employing C18 chromatographic column (4.6 × 150 mm, 5 μm). Acetonitrile was utilized as mobile phase. The flow rate was 1 mL/min and UV detection wavelength of PPa-EG and FSSP was 409 nm.

Redox-responsive prodrug activation was expected to trigger the disintegration of nano-tank, thereby alleviating the ACQ effect. To confirm our hypothesis, the fluorescence spectra of FSSPAO NAs, FSSPA NAs, FCCPA NAs and HSSPA NAs (equal to 5 μg mL^-1^ of PPa) incubated in the media containing 0, 0.5, or 1 mM of DTT at 37 °C for 0, 2 or 4 h. were scanned, respectively. The fluorescence spectra were scanned via multimode microplate reader (Thermo Scientific, USA).

In addition to ACQ relief, redox-responsive NAs would also be expected to improve photodynamic conversion efficiency. Thus, singlet oxygen sensor green (SOSG) testing kits were utilized to detect *in vitro* singlet oxygen generation of NAs. Briefly, PPa Sol, HSSPA NAs, FCCPA NAs, FSSPA NAs, HSSPAO NAs, FCCPAO NAs and FSSPAO NAs (equal to 2 μM of PPa) were incubated with SOSG dilution (2 μM), respectively. Then, the singlet oxygen generation of above samples under laser irradiation (660 nm, 20 mW cm^-2^) for 5 min was tested via multimode microplate reader (Thermo Scientific, USA). To further assess the improved photodynamic conversion efficiency induced by ACQ relief, FSSPO NAs, HSSPAO NAs, FCCPAO NAs and FSSPAO NAs (equal to 2 μM of PPa) mixed with SOSG dilution (2 μM) were incubated with DTT (1 mM) for 4 h. Afterwards, singlet oxygen production of such samples under laser irradiation (660 nm, 20 mW cm^-2^) for 5 min was evaluated by multimode microplate reader (Thermo Scientific, USA).

***In vitro* release patterns of ATO**. Given that ATO is a hydrophobic molecule, 20% of tetrahydrofuran was added into PBS (pH 7.4) to solubilize ATO for evaluating the *in vitro* release patterns of NAs. To verify the redox-sensitive ATO release profiles from FSSPA NAs, FCCPA NAs and HSSPA NAs, 1 mL of NAs (equal to 0.5 μmol mL^-1^ of ATO) was incubated in dialysis bags, which immersed in 30 mL release medium containing 0, 0.2, 0.5, or 1 mM of DTT at 37 °C, respectively. At the preset time points, the accumulative release of ATO were measured by HPLC. To further determine the DTT-responsive drug release under laser irradiation, 1 mL of FSSPA NAs or FCCPA NAs or HSSPA NAs (equal to 0.5 μmol mL^-1^ of ATO) was incubated in dialysis bags, which immersed in 30 mL release medium with laser irradiation (660 nm, 20 mW cm^-2^) for 5 min at 37 °C. At the presupposed time points, the accumulative release of ATO were measured by HPLC. Moreover, to investigate the effect of oxygen loading on ATO release characteristics of binary NAs, 1 mL of FSSPA NAs, FCCPA NAs, HSSPA NAs, FSSPAO NAs, FCCPAO NAs and HSSPAO NAs (equal to 0.5 μmol mL^-1^ of ATO) was incubated in dialysis bags, which immersed in 30 mL release medium containing 1 mM of DTT at 37 °C, respectively. At the preset time points, the accumulative release of ATO were measured by HPLC. Chromatographic separation was carried out employing C18 chromatographic column (4.6 × 150 mm, 5 μm). Acetonitrile and ammonium acetate (0.02 M, pH 3) were utilized as mobile phase at a ratio of 85:15 (v/v). The flow rate was 1 mL min^-1^ and UV detection wavelength of ATO was 254 nm.

**Cellular uptake.** The cellular uptake efficiency of binary NAs was investigated by detecting the intracellular fluorescence intensity of PPa in 4T1 and CT26 cells after incubation with different formulations. Briefly, 4T1 or CT26 cells were seeded in 24-well plates at a density of 5×10^4^ cells/well. After overnight incubation, the 4T1 or CT26 cells were cultured with PPa Sol, FSSPO NAs, HSSPA NAs, FCCPA NAs, FSSPA NAs, HSSPAO NAs, FCCPAO NAs, FSSPAO NAs (equal to 2.5 μg mL^-1^ of PPa) for 1 and 4 h, respectively. Afterwards, the cells were further incubated with Hoechst 33342 and continuously fixed with 4% paraformaldehyde for 10 min. The intracellular fluorescence was observed by confocal laser scanning microscopy (CLSM, C2, Nikon, Japan). To verify the ACQ-induced cellular uptake difference, the actual fluorescence intensity of PPa Sol and NAs after cell disruption was further quantitatively determined using a multimode microplate reader (Thermo Scientific, USA).

**Cellular oxygen consumption suppression.** The mitochondrial damage was evaluated using MitoTracker Green (Mito-G) according to the protocol. Briefly, 4T1 or CT26 cells were seeded in 24-well plates (5 × 10^4^ cells/well). After overnight incubation, the tumor cells were cultured with PBS, ATO Sol, FSSPO NAs, HSSPA NAs, FCCPA NAs, FSSPA NAs, HSSPAO NAs, FCCPAO NAs and FSSPAO NAs (equal to 2 μM of PPa or 1 μM of ATO) for 12 h, respectively. Afterwards, the cells were further incubated with MitoTracker Green (2 μM) for 20 min. Then, the fluorescence of cells was observed by CLSM (C2, Nikon, Japan).

To verify the mechanism of ATO-mediated mitochondrial damage, the activity of mitochondrial complex III in 4T1 and CT26 cells treated with different formulations was further evaluated. Briefly, 4T1 cells were cultured in dishes at a density of 10^6^ cells/dish. After overnight incubation, the 4T1 and CT26 cells were then treated with PBS, FSSPO NAs, ATO Sol, HSSPA NAs, FCCPA NAs, FSSPA NAs, HSSPAO NAs, FCCPAO NAs and FSSPAO NAs (equal to 2 μM of PPa or 1 μM of ATO) for 12 h, respectively. After that, cells were collected to extract mitochondria. Finally, the activity of mitochondrial complex III in 4T1 and CT26 cells was evaluated using the mitochondrial complex ΙII testing kits according to the protocol.

To further detect the efficiency of inhibiting cell oxygen consumption, the 4T1 and CT26 cells were cultured in dishes at a density of 10^6^ cells/dish. After overnight incubation, the cells were then administrated with PBS (pH 7.4), PPa Sol, ATO Sol, FSSPO NAs, HSSPA NAs, FCCPA NAs, FSSPA NAs, HSSPAO NAs, FCCPAO NAs and FSSPAO NAs (equal to 2 μM of PPa or 1 μM of ATO) for 12 h, respectively. Then, liquid paraffin was applied to seal the culture medium for avoiding oxygen exchange, and the real-time oxygen content in cell culture medium was recorded utilizing a dissolved oxygen meter (WLDO-300, Shanghai Water Inst Technology CO. Ltd.).

**Cellular HIF-1α expression.** To investigate the *in vitro* hypoxia relief effect, the hypoxia inducible factor (HIF-1α) expressed in both 4T1 and CT26 cells receiving different formulations was evaluated via immunofluorescence staining assay. Briefly, 4T1 and CT26 cells were seeded in 24-well plates (5 × 10^4^ cells/well) under normoxic and hypoxic environment, respectively. After overnight incubation, the 4T1 and CT26 cells were cultured with PBS, PPa Sol, ATO Sol, FSSPO NAs, FCCPA NAs, FSSPA NAs, HSSPAO NAs, FCCPAO NAs and FSSPAO NAs under a hypoxic environment at equivalent PPa of 2 μM for 12 h, respectively. After that, the 4T1 and CT26 cells cultured with PPa-containing formulations were irradiated with 660 nm laser (20 mW cm^-2^) for 5 min. Untreated cells under normoxic environment were utilized as control. Then, all the tumor cells were fixed with 4% paraformaldehyde, followed by incubating with anti-HIF-1α antibodies (1:200) for 12 h in refrigerator at 4°C. Subsequently, the cells were stained with secondary antibodies at room temperature. After 2 h, the cells were further cultured with Hoechst 33342 for nuclear staining. The fluorescence signals were observed using CLSM (C2, Nikon, Japan). Apart from immunofluorescence staining assay, western blot assay was further used to investigate the expression of HIF-1α, 4T1 and CT26 cells were cultured in culture dishes for 24 h under hypoxic environment. After that, the 4T1 and CT26 cells were cultured with PBS, HSSPAO NAs, FCCPAO NAs and FSSPAO NAs under hypoxic environment at equivalent PPa of 2 μM for 12 h, respectively. Then, the proteins of HIF-1α were obtained and analyzed by western blot. In addition, PBS (pH 7.4)-treated 4T1 and CT26 cells under normoxic environment were used as control.

***In vitro* PDT efficacy.** The ROS generation in 4T1 and CT26 cells treated with different formulations was detected using a DCFH-DA probe. Briefly, 4T1 and CT26 cells were seeded into 24-well plates at a density of 5 × 10^4^ cells/well under normoxic or hypoxic condition. After 12 h incubation, the tumor cells were incubated with PBS, PPa Sol, FSSPO NAs, FCCPA NAs, FSSPA NAs, HSSPAO NAs, FCCPAO NAs and FSSPAO NAs (equal to 200 nM of PPa) for 12 h under normoxic or hypoxic condition. After that, the cells were further cultured DCFH-DA probe (20 μM) for 30 min. Subsequently, the cells were irradiated at 660 nm (20 mW cm^-2^) for 5 min. Finally, the cellular fluorescence intensity was observed by an inverted microscope (Nikon Corp., Tokyo, Japan).

Moreover, the effects of FSSPA NAs to normal cells was evaluated via MTT assay. Briefly, L929 cells were seeded in 96-well plates (2 × 10^3^ cells/well) for 12 h. Then, the cells were further cultured with fresh media containing gradient concentrations of FSSPA NAs for 48 h. Untreated cells were used as control. Later, 20 μL of MTT solution was added for another 4 h, after which the medium was discarded and dissolved in DMSO. The absorbance of the plates was recorded on a multimode microplate reader (Thermo Scientific, USA) at 490 nm.

The dark toxicity of ATO Sol and PPa Sol was further evaluated using MTT assay. Briefly, 4T1 and CT26 cells were seeded in 96-well plates (2 × 10^3^ cells/well) for 12 h under normoxic or hypoxic condition. Then, the cells were further cultured with fresh media containing gradient concentrations of ATO Sol or PPa Sol for 48 h in normoxic and hypoxic environment, respectively. Untreated cells were used as control. Later, 20 μL of MTT solution was added for another 4 h, after which the medium was discarded and dissolved in DMSO. The absorbance of the plates was recorded on a multimode microplate reader (Thermo Scientific, USA) at 490 nm. Moreover, the *in vitro* cytotoxicity of NAs with laser irradiation (660 nm, 20 mW cm^-2^, 5 min) against 4T1 and CT26 cells under normoxic or hypoxic conditions was also evaluated similarly to above process, except that ATO Sol or PPa Sol was replaced by NAs, respectively. The process of light irradiation at 660 nm (20 mW cm^-2^, 5 min) was added after 12 h of culture.

**Animal studies.** All animal procedures were performed in accordance with the Guidelines for Care and Use of Laboratory Animals of Shenyang Pharmaceutical University and approved by the Animal Ethics Committee of Shenyang Pharmaceutical University.

***In vivo* pharmacokinetics.** Sprague-Dawley rats (200-220 g) were applied to investigate the pharmacokinetic profiles of (n = 5). PPa Sol, FSSPO NAs, FSSPA NAs, HSSPAO NAs, FCCPAO NAs and FSSPAO NAs were intravenously administrated at an equivalent PPa dose of 2 mg kg^-1^. At preset time intervals (0.033, 0.083, 0.25, 0.5, 1, 2, 4, 8 and 12 h), blood samples were collected and centrifuged (1 × 10^4^ rpm, 3 min) to obtain plasma. Finally, the concentrations of PPa in plasma were detected via multimode microplate reader (Thermo Scientific, USA).

***Ex vivo* biodistribution.** The *ex vivo* biodistribution of the nanoassemblies was evaluated in 4T1 tumor-bearing BALB/c mice. When the volume of subcutaneous 4T1 tumors reached around 500 mm^3^, PPa Sol, FSSPO NAs, FSSPA NAs, HSSPAO NAs, FCCPAO NAs and FSSPAO NAs were intravenously administered at an equivalent PPa dose of 4 mg kg^-1^ (n = 4). At certain time intervals (4, 12 and 24 h), the mice were sacrificed, and the major organs and tumors were isolated and collected. The major organs and tumors were imaged by *in vivo* imaging system (IVIS Lumina Series III).

***In vivo* photoacoustic imaging.** The ability of oxygen nano-tank in enhancing tumor oxygenation was evaluated in 4T1 tumor-bearing BALB/c mice. When the tumor volume was approximately 500 mm^3^, saline, FSSPA NAs, FSSPO NAs and FSSPAO NAs were intravenously administered (equal to 2.2 mg kg^-1^ 1H,1H-perfluorohexylamine or 4 mg kg^-1^ of PPa). At certain time intervals, the 4T1 tumor-bearing BALB/c mice were anesthetized with a small animal gas anesthesia machine (Shanghai Yuyan Instruments Co.,Ltd.), and the oxyhemoglobin signals of 4T1 tumor-bearing BALB/c mice at 1064 nm were monitored using a PA imaging system (LOIS 3D TomoWave Laboratories, USA).

***Ex vivo* evaluation of hypoxia relief.** The hypoxia status was also evaluated in 4T1 tumor-bearing BALB/c mice by the Hypoxyprobe-1 plus kit (Hypoxyprobe Inc.). When the volume of 4T1 tumors reached around 500 mm^3^, saline, FSSPA NAs, FSSPO NAs and FSSPAO NAs (equal to 2.2 mg kg^-1^ 1H,1H-perfluorohexylamine or 4 mg kg^-1^ of PPa) were intravenously administered. After 12 h, 4T1 tumor-bearing BALB/c mice were intraperitoneally injected with pimonidazole hydrochloride (60 mg/kg). After 30 min, the tumors were collected, immersed in O.C.T. (Leica, Germany) and frozen in liquid nitrogen. Finally, the immunofluorescence imaging of tumor tissues was observed by CLSM (C2, Nikon, Japan).

***In vivo* photodynamic tumor eradication.** The closed-loop hypoxia alleviation-driven photodynamic tumor eradication was also evaluated in 4T1 tumor-bearing BALB/c mice and CT26 tumor-bearing BALB/c mice, respectively. After the tumor volume was about 150 mm^3^, the mice were randomly divided into 9 groups: Saline, ATO Sol, PPa Sol/L, ATO/PPa mixture/L, FSSPO NAs/L, FSSPA NAs/L, HSSPAO NAs/L, FCCPAO NAs/L and FSSPAO NAs/L (n = 5). The formulations (equal to 1.4 mg kg^-1^ of ATO or 4 mg kg^-1^ of PPa) via the tail vein at intervals of 2 days for five injections totally. According to the biodistribution results, the mice in PPa Sol, PPa/ATO mixture and HSSPAO NAs-treated groups were exposed to laser (660 nm, 20 W cm^-2^) for 5 min at 4 h post-administration, and FSSPO NAs, FSSPA NAs, FCCPAO NAs and FSSPAO NAs-treated groups were received to laser (660 nm, 20 W cm^-2^) for 5 min at 12 h post administration. During the whole period of treatment, body weights and tumor sizes of the mice were monitored and calculated every day. The tumor volumes were calculated by following formula: V = width^2^ × length/2. At the end of the therapy, the mice were sacrificed, and the serum samples were also collected for blood routine assessment, and hepatic and renal function analysis. In addition, the haemolysis assay was conducted via incubating the red blood cells of mice with water, saline, ATO Sol, PPa Sol, ATO/PPa mixture, FSSPO NAs, FSSPA NAs, HSSPAO NAs, FCCPAO NAs and FSSPAO NAs at 37 °C for 4 h, and measuring the absorbance at 540 nm of the released hemoglobins. Finally, the main organs (heart, liver, spleen, lung and kidney) and tumors were excised for pathological analysis.

**Statistical analysis.** All the data were expressed as mean value ± standard deviation (SD). One-way analysis of variance (ANOVA) or t-test was utilized to identify the significant differences between groups. No significant (n.s.) differences were defined as p > 0.005, while statistical differences were considered as *p < 0.05, **p < 0.01, ***p < 0.001 and ****p < 0.0001.

**Supplementary Figures**


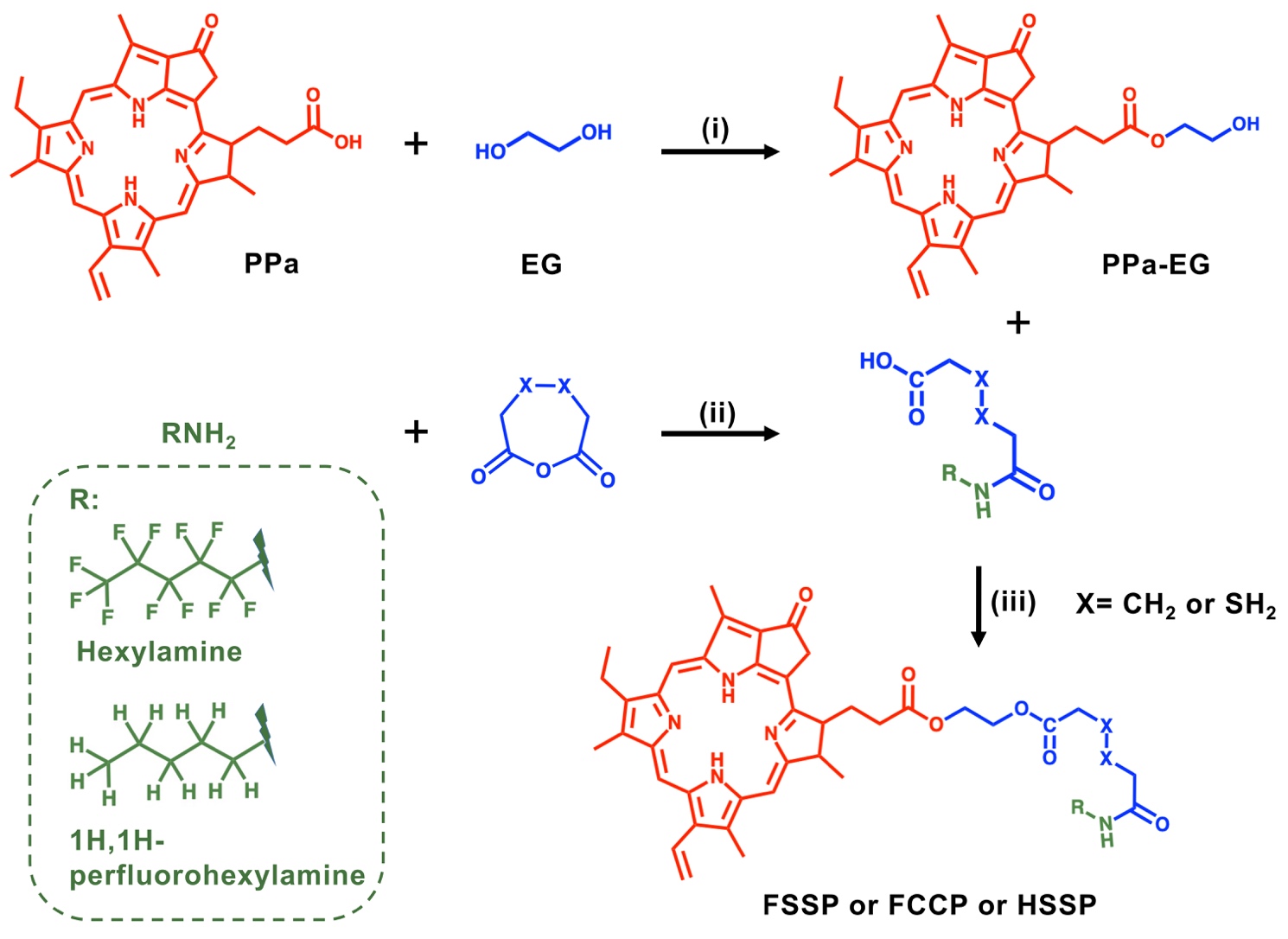


**Figure S1.** Synthetic routes of prodrugs. (i) EDCI, HOBt, DMAP, 25 °C; (ii) DMAP, r.t.; (iii) EDCI, HOBt, DMAP, 25 °C. PPa, pyropheophorbide a; EG, ethylene glycol.

**
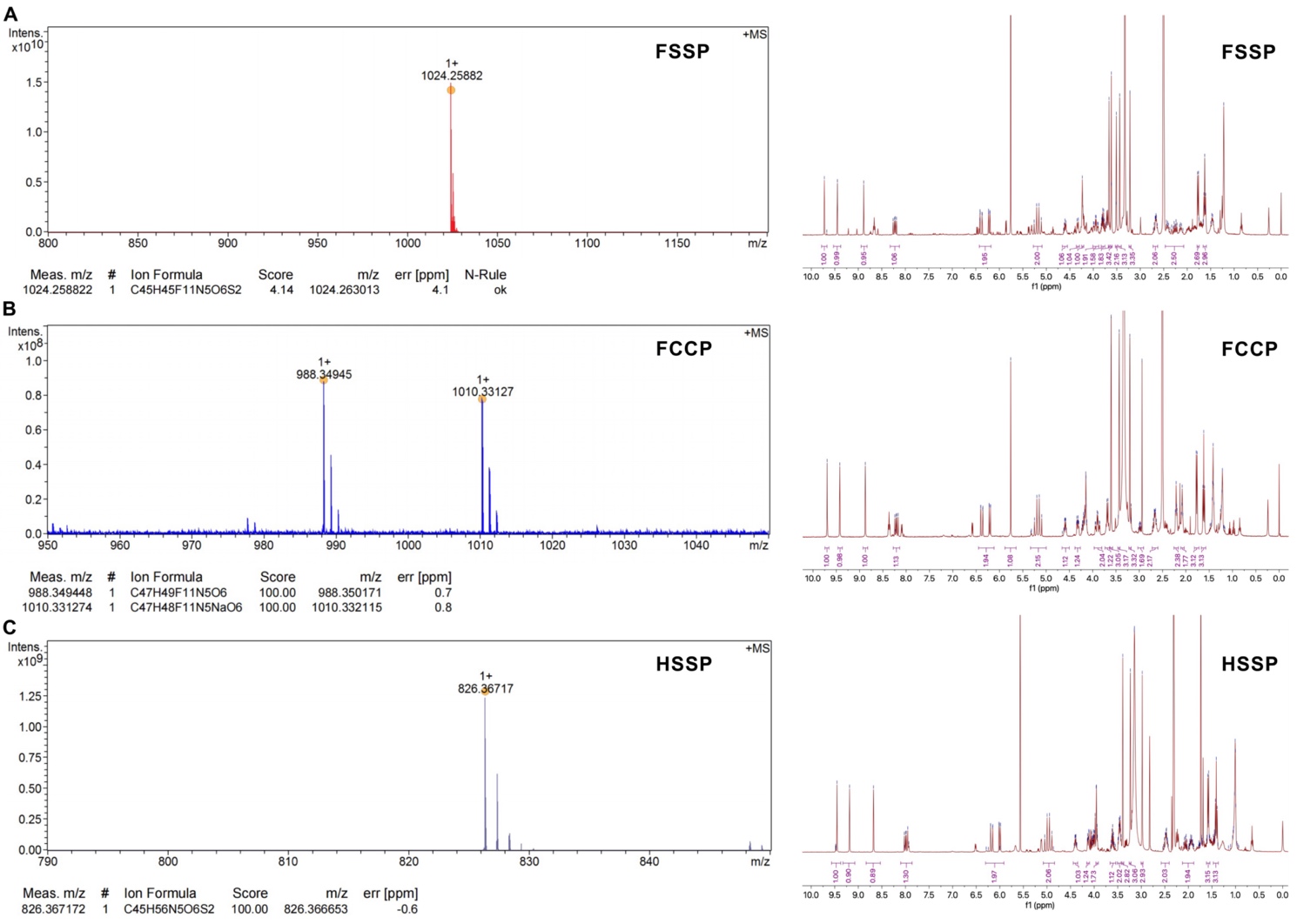
**

**Figure S2.** Mass spectra and ^1^H NMR spectra of FSSP, FCCP and HSSP. (A) MS (ESI) m/z for C_45_H_44_F_11_N_5_O_6_S_2_ [M+H]^+^: 1024.26. ^1^H NMR: δ: 9.72 (s, meso-1H), 9.45 (s, 1H, meso-H), 8.89 (s, 1H, meso-H), 8.26 (m, 1H, H), 6.20-6.41 (m, 2H, H), 5.11-5.26 (dd, 2H, H), 4.58 (m, 1H, H), 4.34 (dd, 1H, H), 3.78 (q, 2H, CH_2_), 3.66 (s, 3H, CH_3_), 3.44 (s, 3H, CH_3_), 3.22 (s, 3H, CH_3_), 2.66-2.68 (m, 2H, CH_2_), 2.24-2.45 (m, 2H, CH_2_), 1.76 (d, 3H, CH_3_), 1.63 (t, 3H, CH_3_). (B) MS (ESI) m/z for C_47_H_49_F_11_N_5_O_6_ [M+H]^+^: 988.35 and C_47_H_48_F_11_N_5_NaO_6_ [M+Na]^+^: 1010.33. ^1^H NMR: δ: 9.70 (s, meso-1H), 9.42 (s, 1H, meso-H), 8.88 (s, 1H, meso-H), 8.20 (m, 1H, H), 6.19-6.40 (m, 2H, H), 5.10-5.76 (dd, 2H, H), 4.57 (m, 1H, H), 4.32 (dd, 1H, H), 3.89 (q, 2H, CH_2_), 3.45 (s, 3H, CH_3_), 3.20 (s, 3H, CH_3_), 3.18 (s, 3H, CH_3_), 2.65-2.67 (m, 2H, CH_2_), 1.78 (d, 3H, CH_3_), 1.64 (t, 3H, CH_3_). (C) MS (ESI) m/z for C_45_H_56_N_5_O_6_S_2_ [M+H]^+^: 826.36. ^1^H NMR: 9.45 (s, meso-1H), 9.19 (s, 1H, meso-H), 8.68 (s, 1H, meso-H), 7.95-8.03 (m, 1H, H), 5.99-6.20 (m, 2H, H), 4.90-5.00 (dd, 2H, H), 4.16 (m, 1H, H), 4.14 (dd, 1H, H), 3.98 (q, 2H, CH_2_), 3.23 (s, 3H, CH_3_), 3.15 (s, 3H, CH_3_), 2.98 (s, 3H, CH_3_), 2.46-2.49 (m, 2H, CH_2_), 1.83-2.15 (m, 2H, CH_2_), 1.58 (d, 3H, CH_3_), 1.43 (t, 3H, CH_3_).


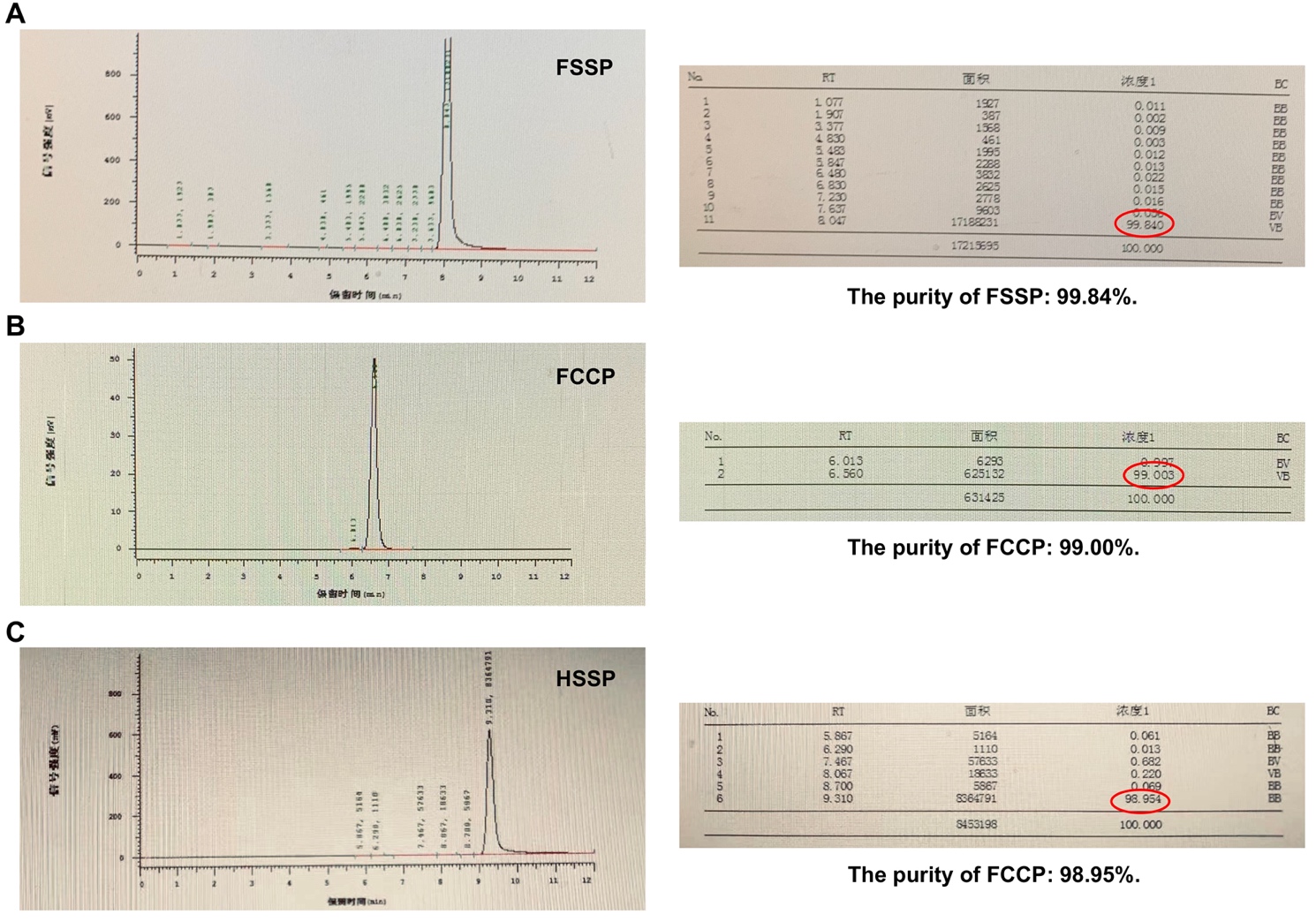


**Figure S3.** The purity of PPa, FSSP, FCCP and HSSP.


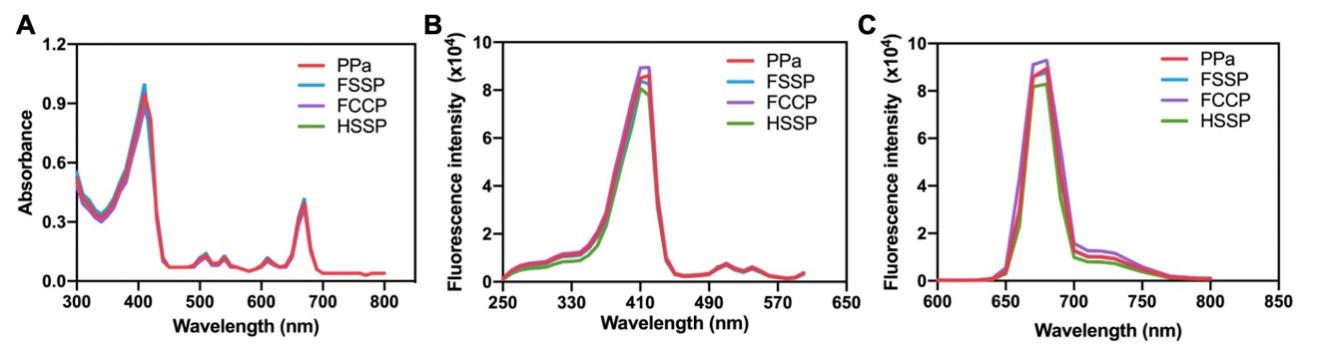


**Figure S4.** Ultraviolet and fluorescence spectra of PPa, FSSP, FCCP and HSSP. (**A**) Ultraviolet spectra at 300-800 nm. (**B**) Excitation spectra at a fixed emission of 415 nm. (**C**) Emission spectra at a fixed excitation of 675 nm.


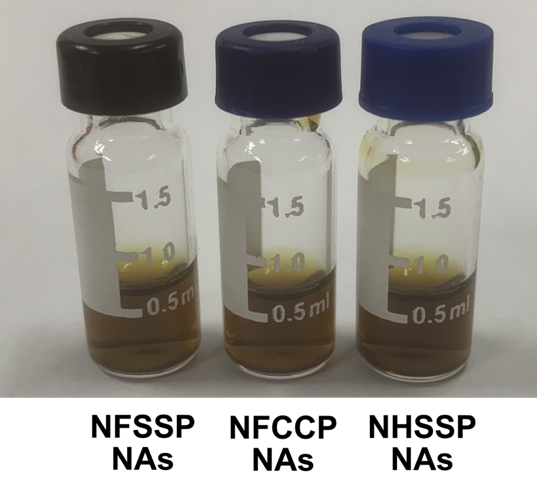


**Figure S5.** Appearance of non-PEGylated prodrug nanoassemblies.


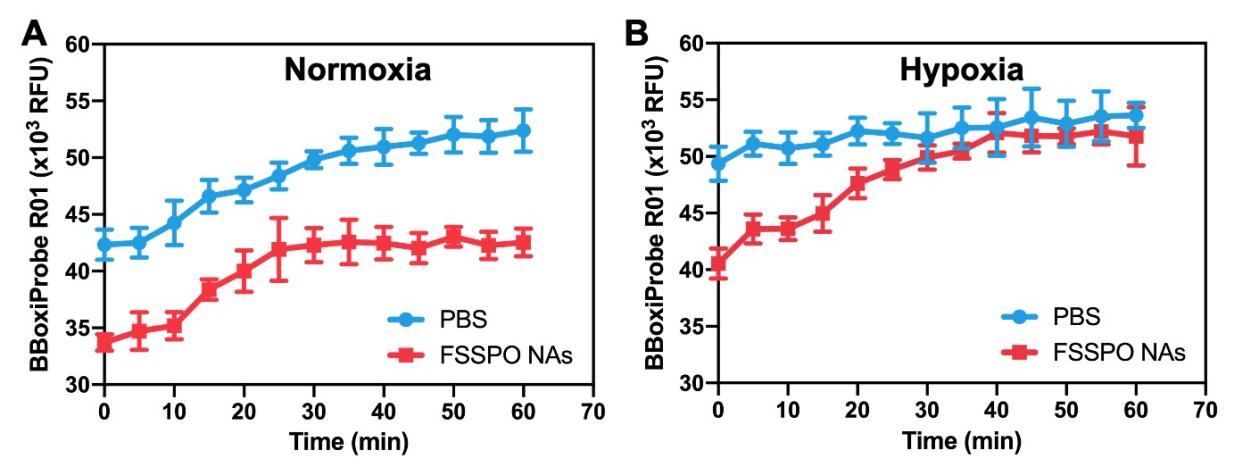


**Figure S6.** Fluorescence of the oxygen probe in CT26 cells under normoxic and hypoxic conditions within 60 min (n = 3).


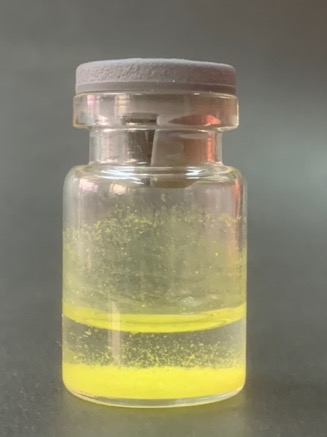


**Figure S7.** Appearance photos of ATO dispersion.


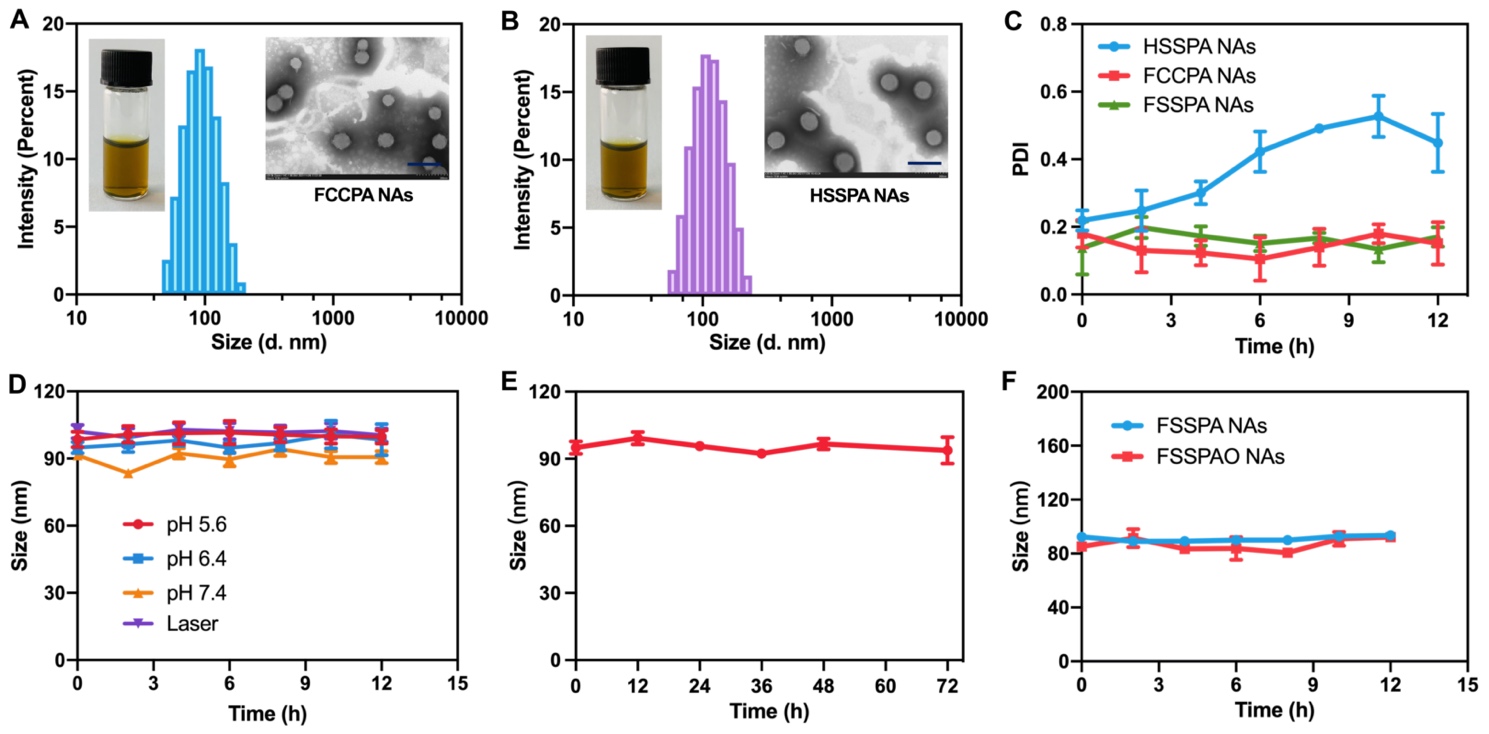


**Figure S8.** (**A**) Appearance photos, particle size distribution profiles and TEM images of FCCPA NAs (scale bar represents 200 nm). (**B**) Appearance photos, particle size distribution profiles and TEM images of HSSPA NAs (scale bar represents 200 nm). (**C**) PDI of three co-assembled NAs incubated in PBS (pH 7.4) containing 10% FBS (n=3). (**D**) Colloidal stability of FSSPA NAs under PBS (pH 5.6, 6.4 and 7.4) incubation and laser irradiation, respectively (n=3). (**E**) Colloidal stability of FSSPA NAs under PBS (pH 7.4) within 72 h (n=3). (**F**) Colloidal stability of FSSPA NAs and FSSPAO NAs (n=3).


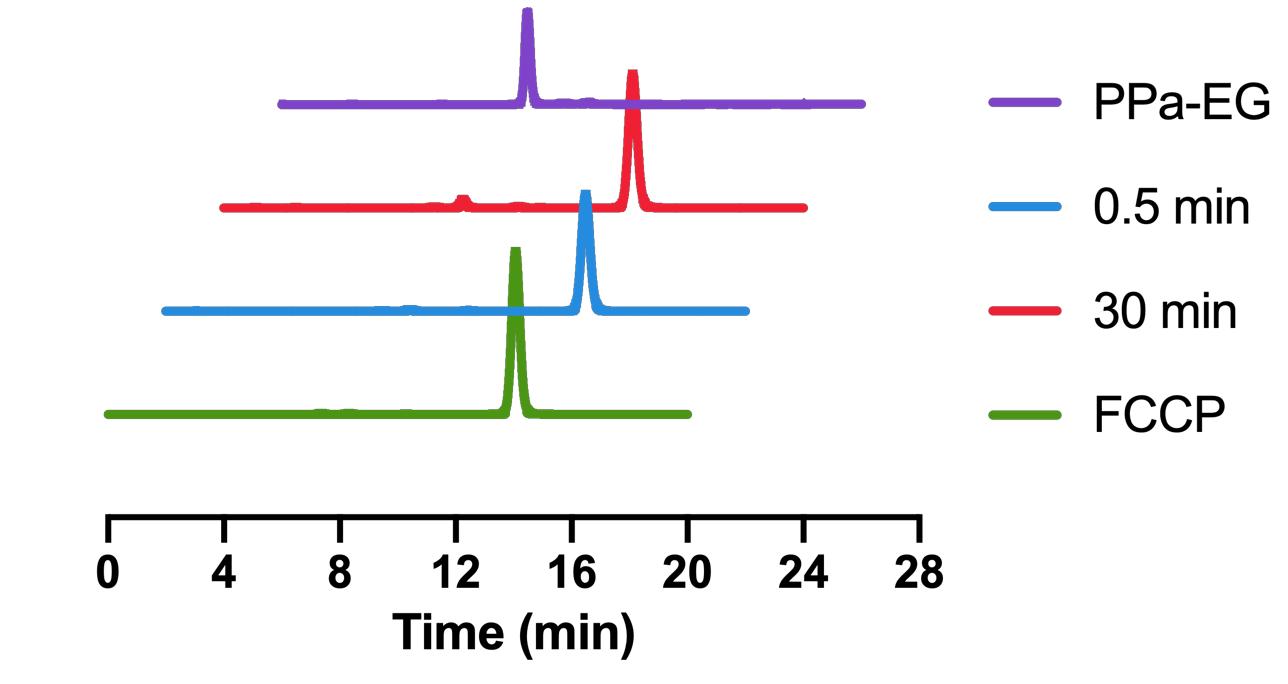


**Figure S9.** DTT-responsive degradation of FCCP prodrug into PPa-EG at different incubation time using HPLC analysis.


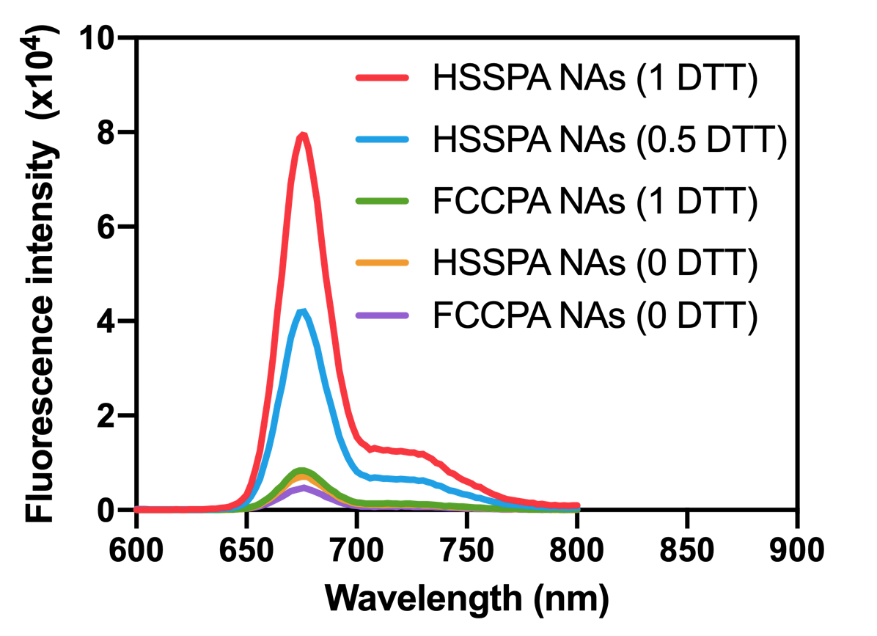


**Figure S10.** Excitation spectral changes of HSSPA NAs and FCCPA NAs when incubated in medium with various concentrations (0, 0.5 and 1mM) of DTT for 4 h.


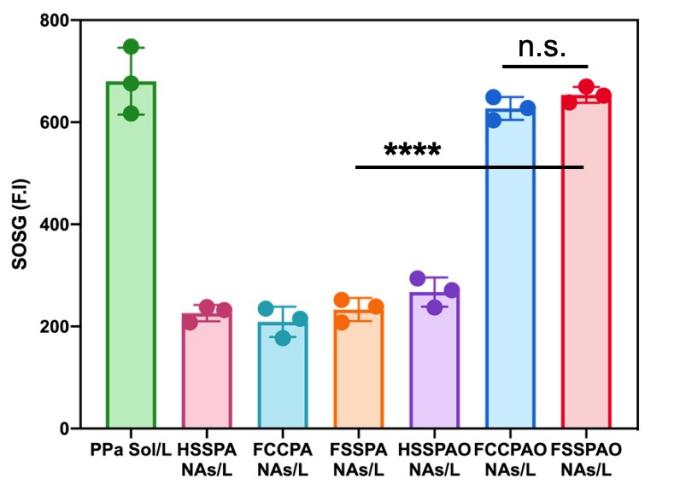


**Figure S11.** The SOSG fluorescence signals of PPa Sol, HSSPA NAs, FCCPA NAs and FSSPA NAs, HSSPAO NAs, FCCPAO NAs and FSSPAO NAs when incubated in medium with laser irradiation (n=3). The “L” represents laser irradiation (660 nm, 20 mW cm^-2^, 5 min).


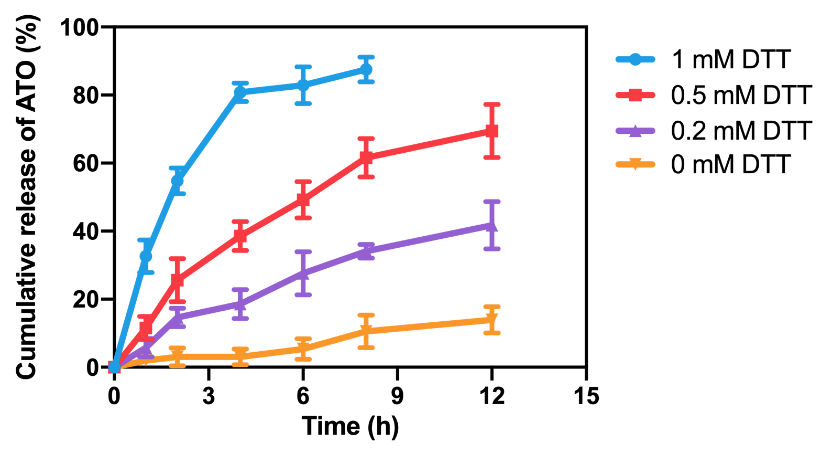


**Figure S12.** ATO release from HSSPA NAs in the presence of various concentrations of DTT (n=3).


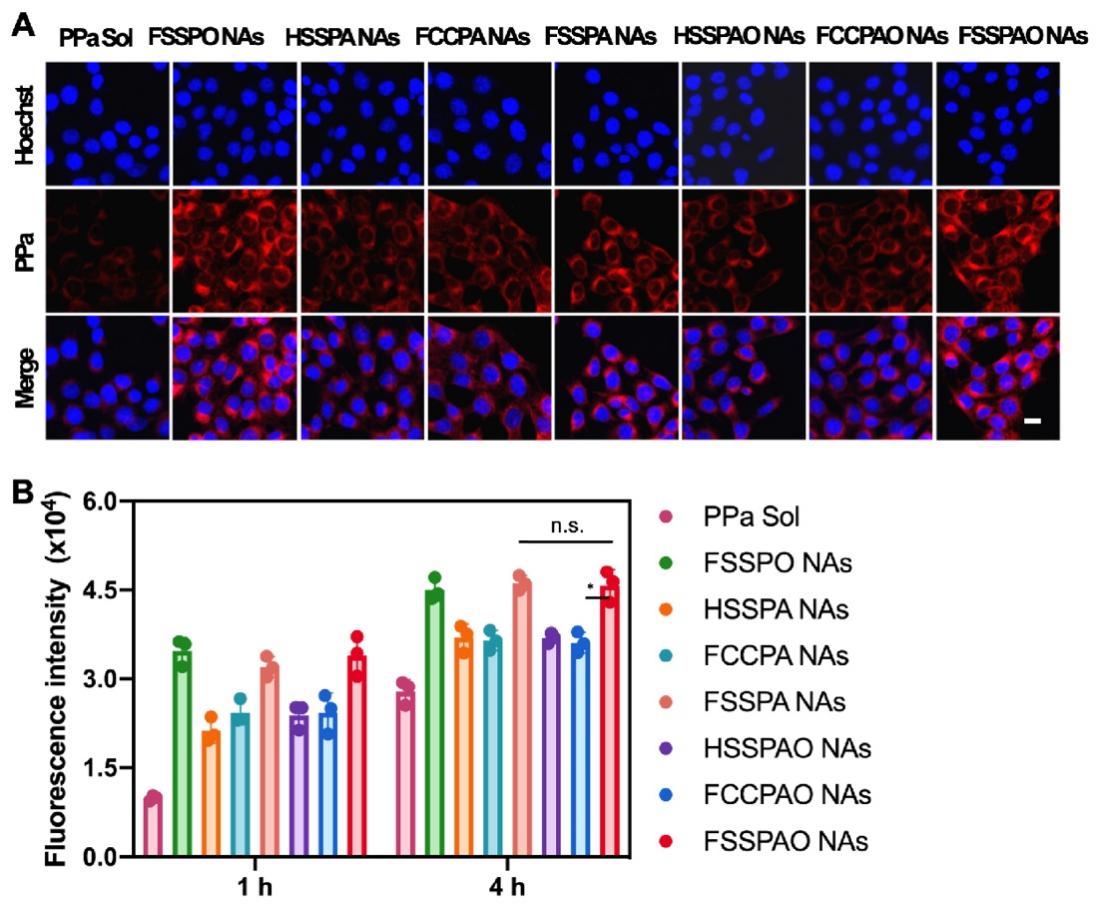


**Figure S13.** (**A**) Cellular uptake in 4T1 cells at 1 h (Scale bar: 10 μm). (**B**) Quantitative analysis of Figure S13A and Figure 4A by using Image J software.


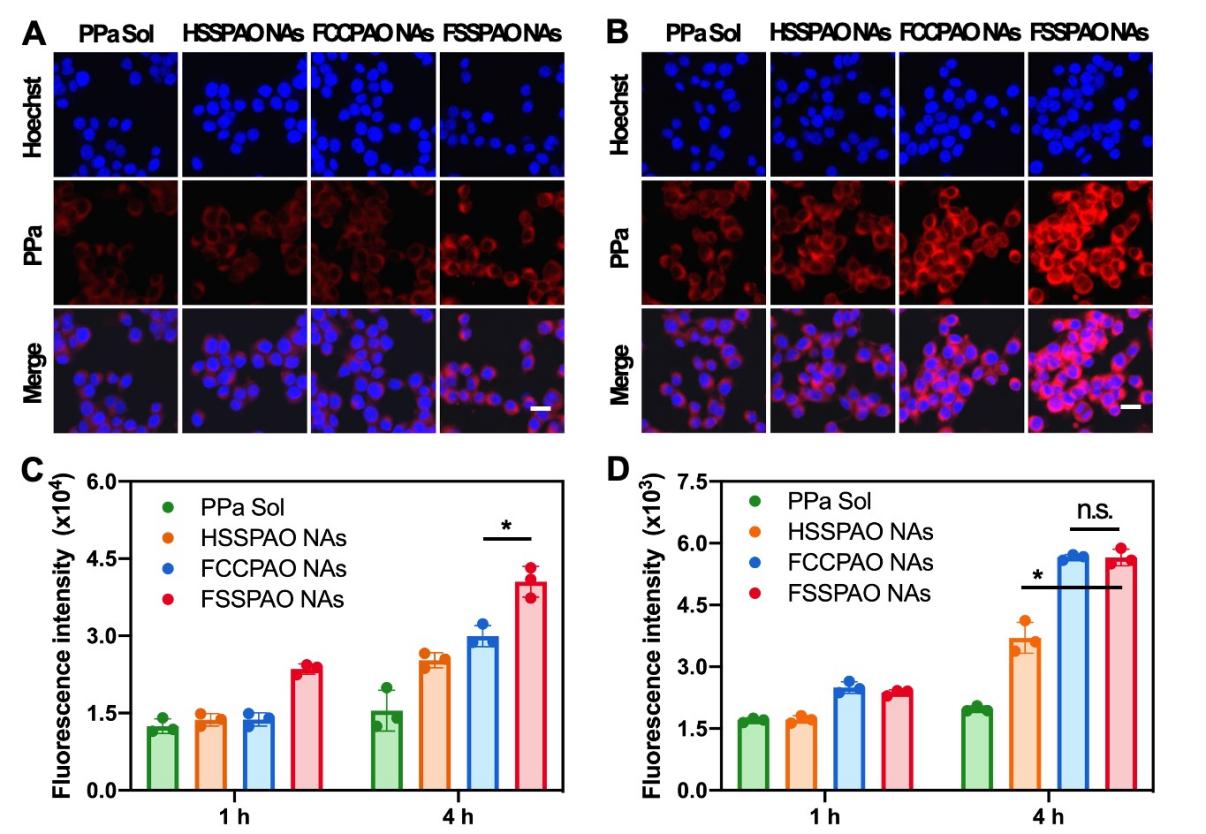


**Figure S14.** (**A**) Cellular uptake in CT26 cells at 1 h (Scale bar: 10 μm). (**B**) Cellular uptake in CT26 cells at 4 h (Scale bar: 10 μm). (**C**) Quantitative analysis of Figure S14A-B by using Image J software. (**D**) Quantitative analysis of cellular uptake in CT26 cells via a multimode microplate reader (Thermo Scientific, USA).


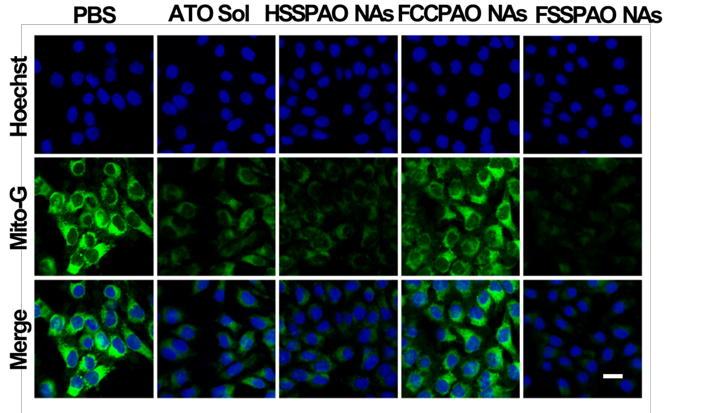


**Figure S15.** CLSM images of CT26 cells after various formulations treatment, then stained by Mito-G (Scale bar: 10 μm).


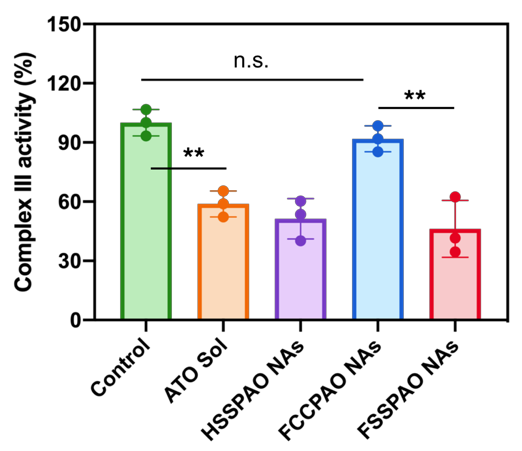


**Figure S16.** Relative activity of mitochondrial complex III in CT26 cells.


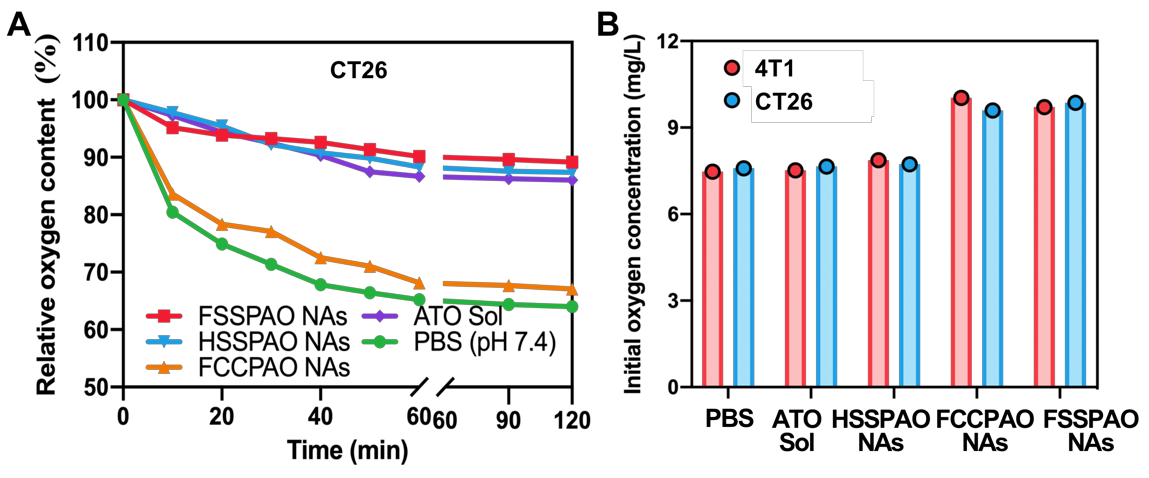


**Figure S17. (A**) Relative oxygen content of CT26 cells after cultured with various formulations. (**B**) Initial oxygen concentration of 4T1 and CT26 cells treated with various formulations.


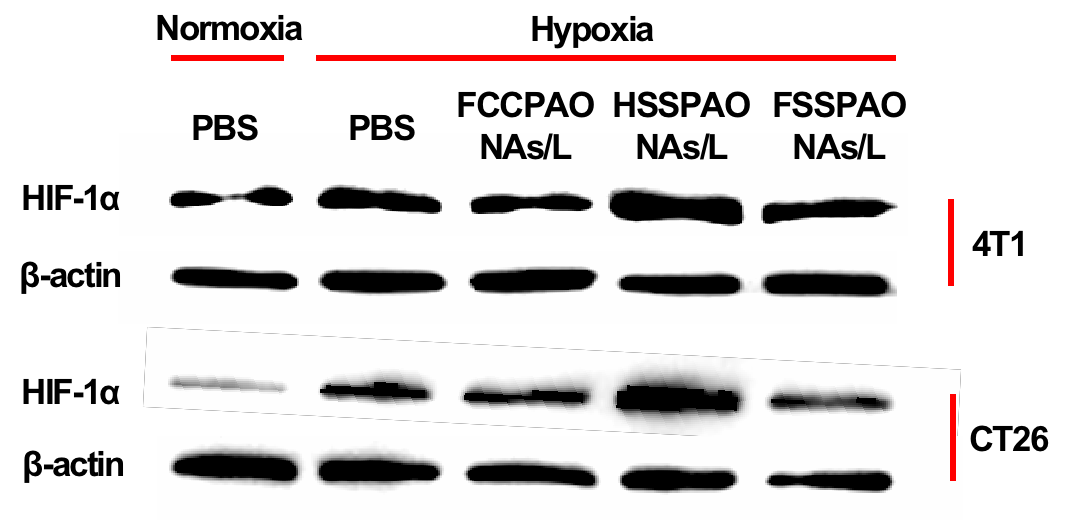


**Figure S18.** Western blot analysis of HIF-1α proteins in 4T1 and CT26 cells under normoxic state and after incubation with different formulations under hypoxic state. The “L” represents laser irradiation (660 nm, 20 mW cm^-2^, 5 min).


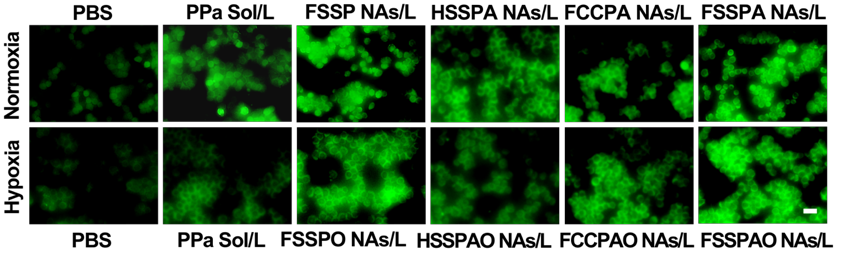


**Figure S19.** Cellular ROS generation in CT26 cells after incubation with various formulations with laser irradiation (660 nm, 20 mW cm^-2^, 5 min) under normoxic and hypoxic state, respectively (Scale bar: 20 μm). The “L” represents laser irradiation (660 nm, 20 mW cm^-2^, 5 min).

**
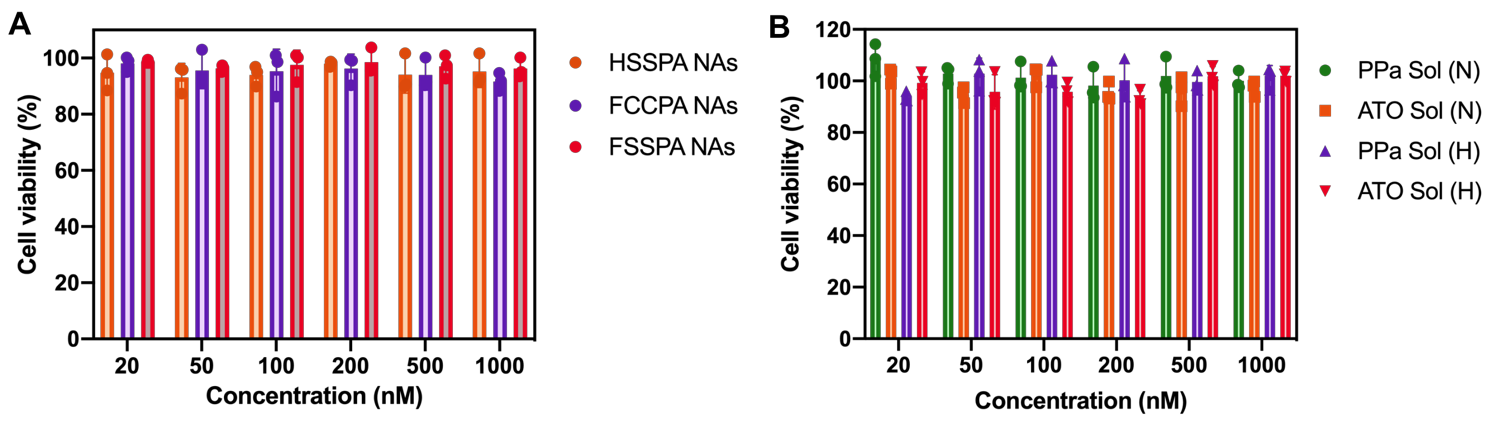
**

**Figure S20.** (**A**) Cytotoxicity of HSSPA NAs, FCCPA NAs and FSSPA NAs against L929 cells. (**B**) Cytotoxicity of PPa Sol without laser irradiation and ATO Sol against 4T1 cells (“N” represents normoxia; and “H” represents hypoxia).

**
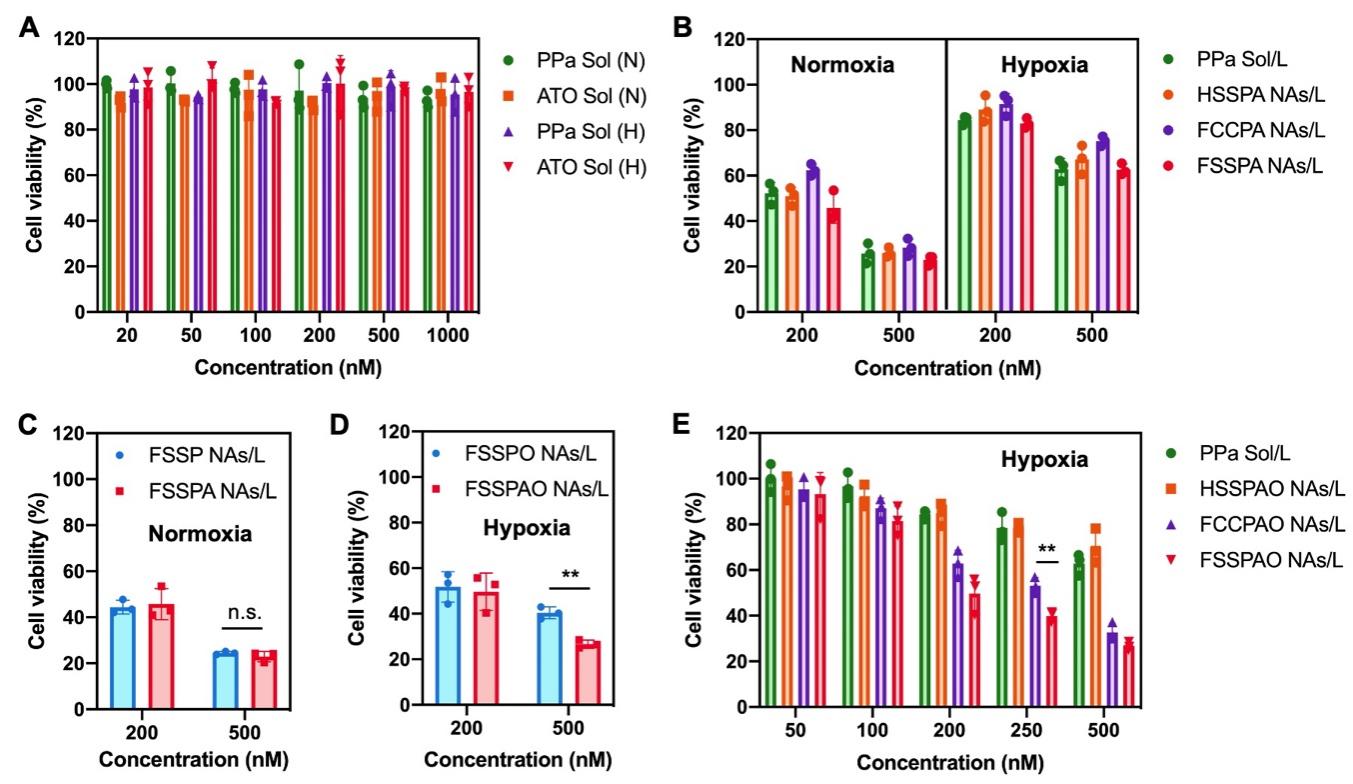
**

**Figure S21.** (**A**) Cytotoxicity of PPa Sol without laser irradiation and ATO Sol in CT26 cells (“N” represents normoxia; and “H” represents hypoxia). (**B**) Cytotoxicity of PPa Sol/L, HSSPA NAs/L, FCCPA NAs/L and FSSPA NAs/L in CT26 cells under normoxic and hypoxic state. (**C**) Cytotoxicity of FSSP NAs/L and FSSPA NAs/L in CT26 cells under normoxic state. (**D**) Cytotoxicity of FSSPO NAs/L and FSSPAO NAs/L in CT26 cells under hypoxic state. (**E**) Cytotoxicity of PPa Sol, HSSPAO NAs, FCCPAO NAs and FSSPAO NAs with laser irradiation (660 nm, 20 mW cm^-2^, 5 min) in CT26 cells under hypoxic state. The “L” represents laser irradiation (660 nm, 20 mW cm^-2^, 5 min).


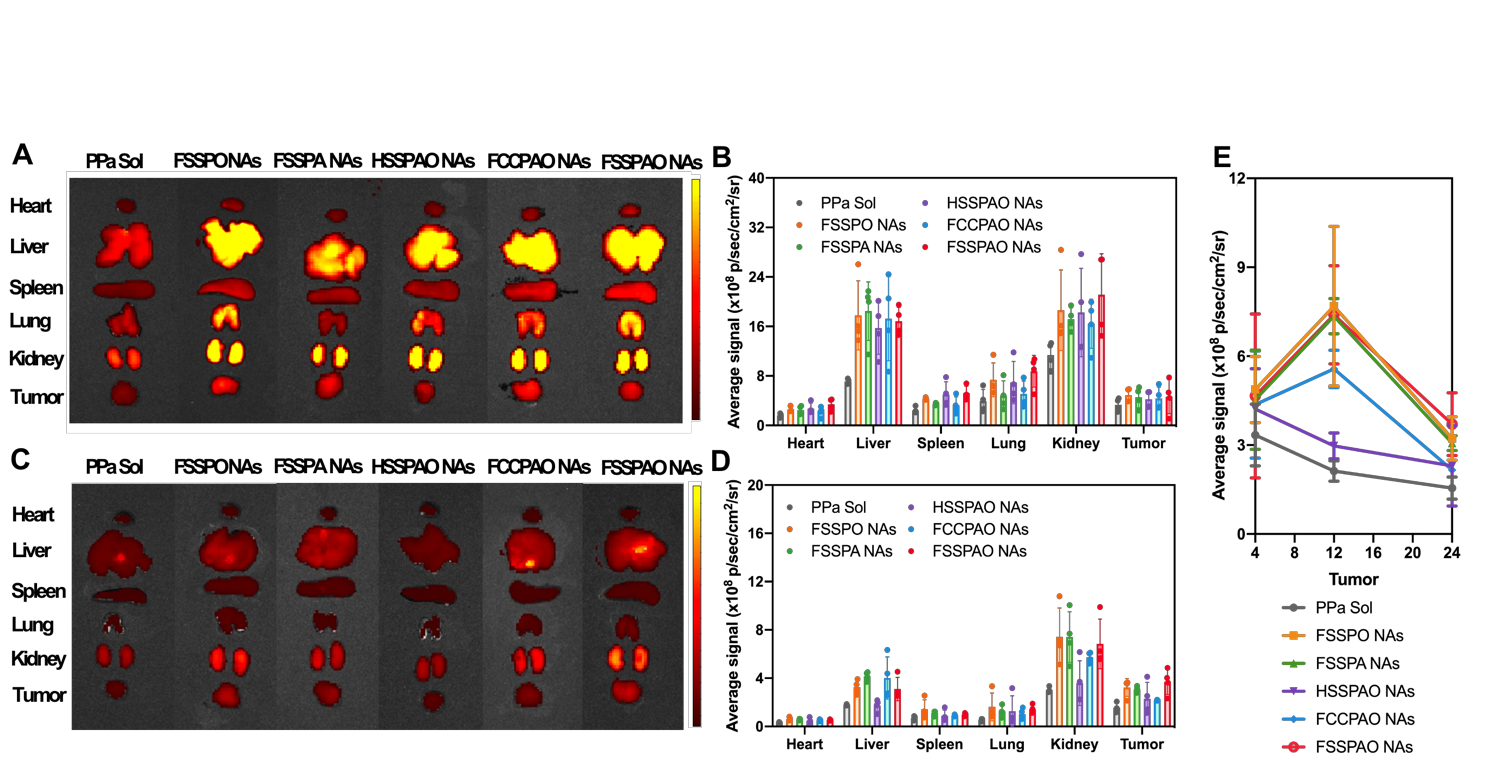


**Figure S22.** (**A**) Fluorescent imaging of organs and tumors in 4T1 tumor-bearing BALB/c mice at 4 h post administration. (**B**) Fluorescence intensity results of organs and tumors in 4T1 tumor-bearing BALB/c mice at 4 h (n = 4). Data were analyzed by IVIS spectrum small-animal *in vivo* imaging system. (**C**) Fluorescent imaging of organs and tumors in 4T1 tumor-bearing BALB/c mice at 24 h post administration. (**D**) Fluorescence intensity results of organs and tumors in 4T1 tumor-bearing BALB/c mice at 24 h (n = 4). Data were analyzed by IVIS spectrum small-animal *in vivo* imaging system. (**E**) The curve of fluorescence intensity of tumor sites at 4 h,12 h and 24 h after injection of PPa Sol, FSSPO NAs, FSSPA NAs, HSSPAO NAs, FCCPAO NAs and FSSPAO NAs.


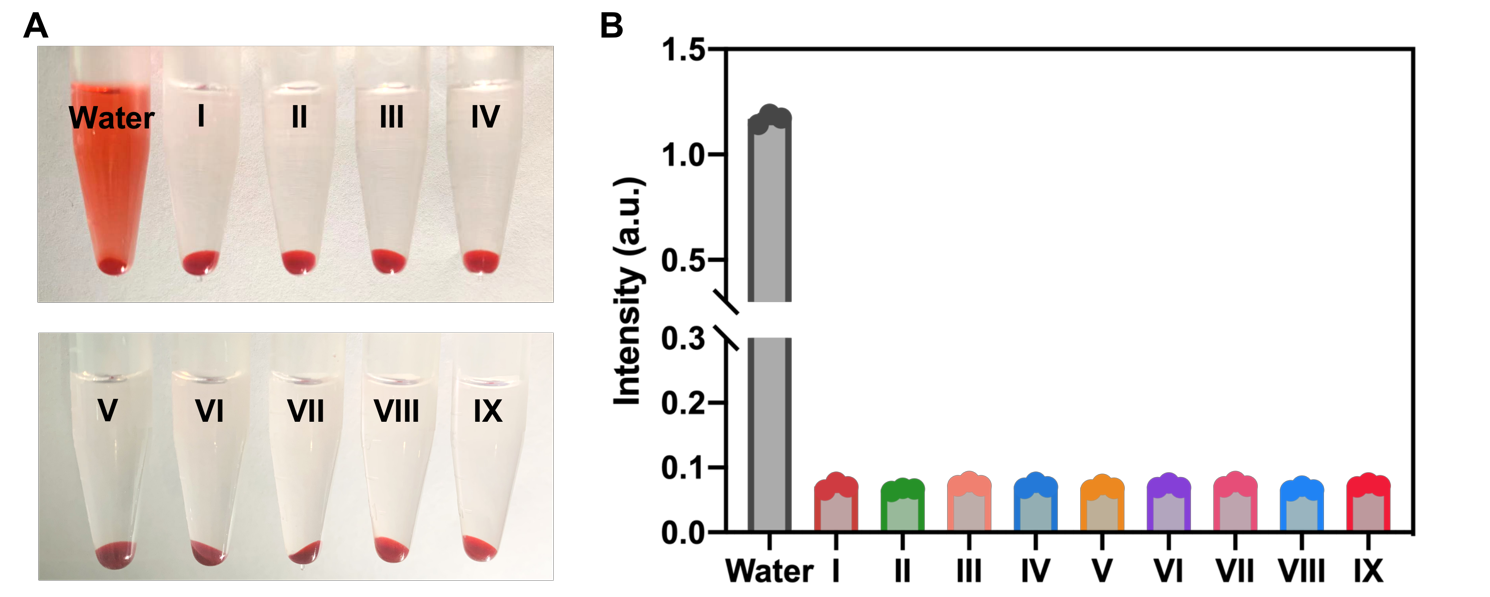


**Figure S23.** (**A**) Hemolysis image of water and different formulations. (**B**) The absorbance at 540 nm of the released hemoglobins. The I, II, III, IV, V, VI, VII, VIII and IX represent saline, ATO Sol, PPa Sol, ATO/PPa mixture, FSSPO NAs, FSSPA NAs, HSSPAO NAs, FCCPAO NAs and FSSPAO NAs, respectively.


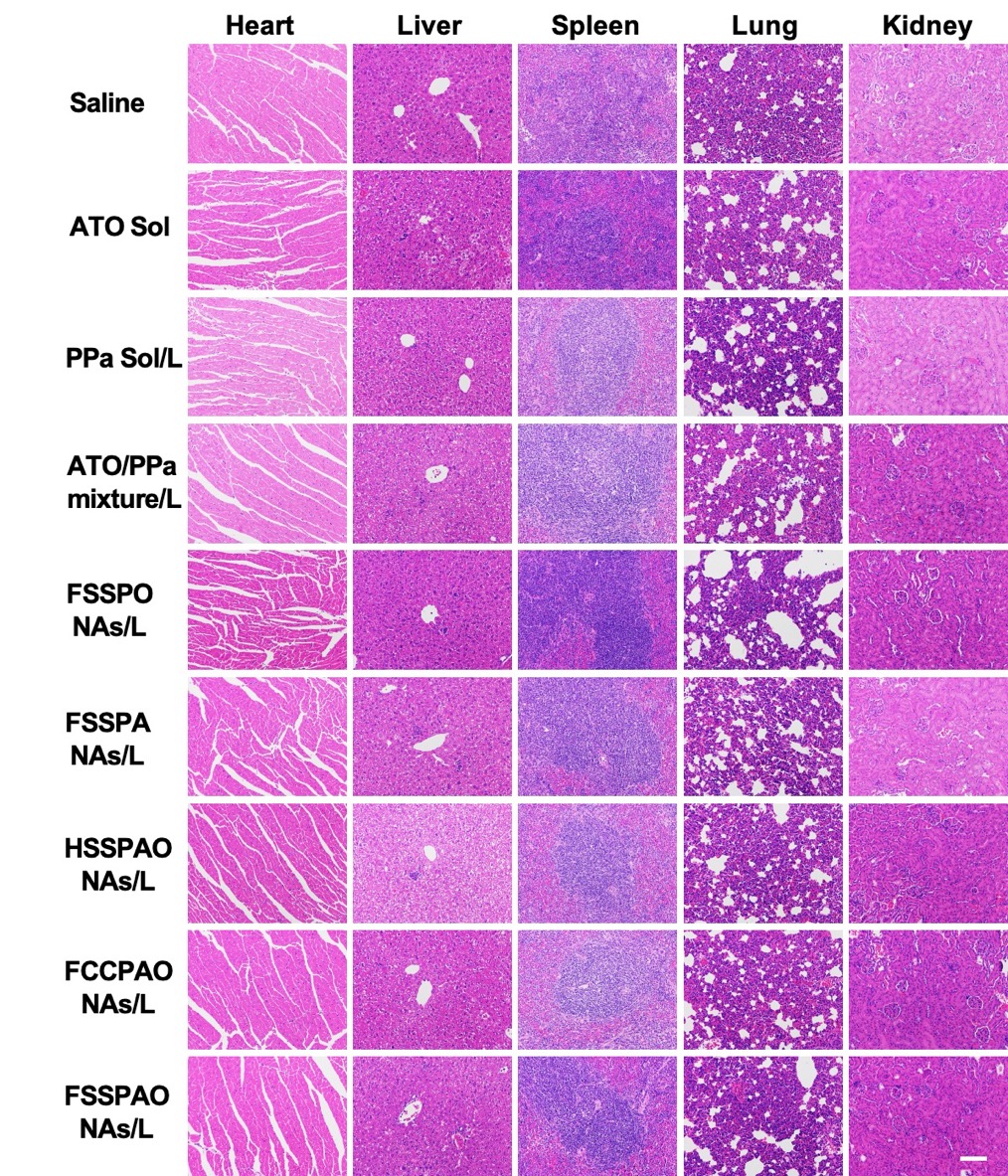


**Figure S24.** H&E staining of the major organs of mice bearing 4T1 xenograft breast tumors after treatments. Scale bar represents 100 μm.


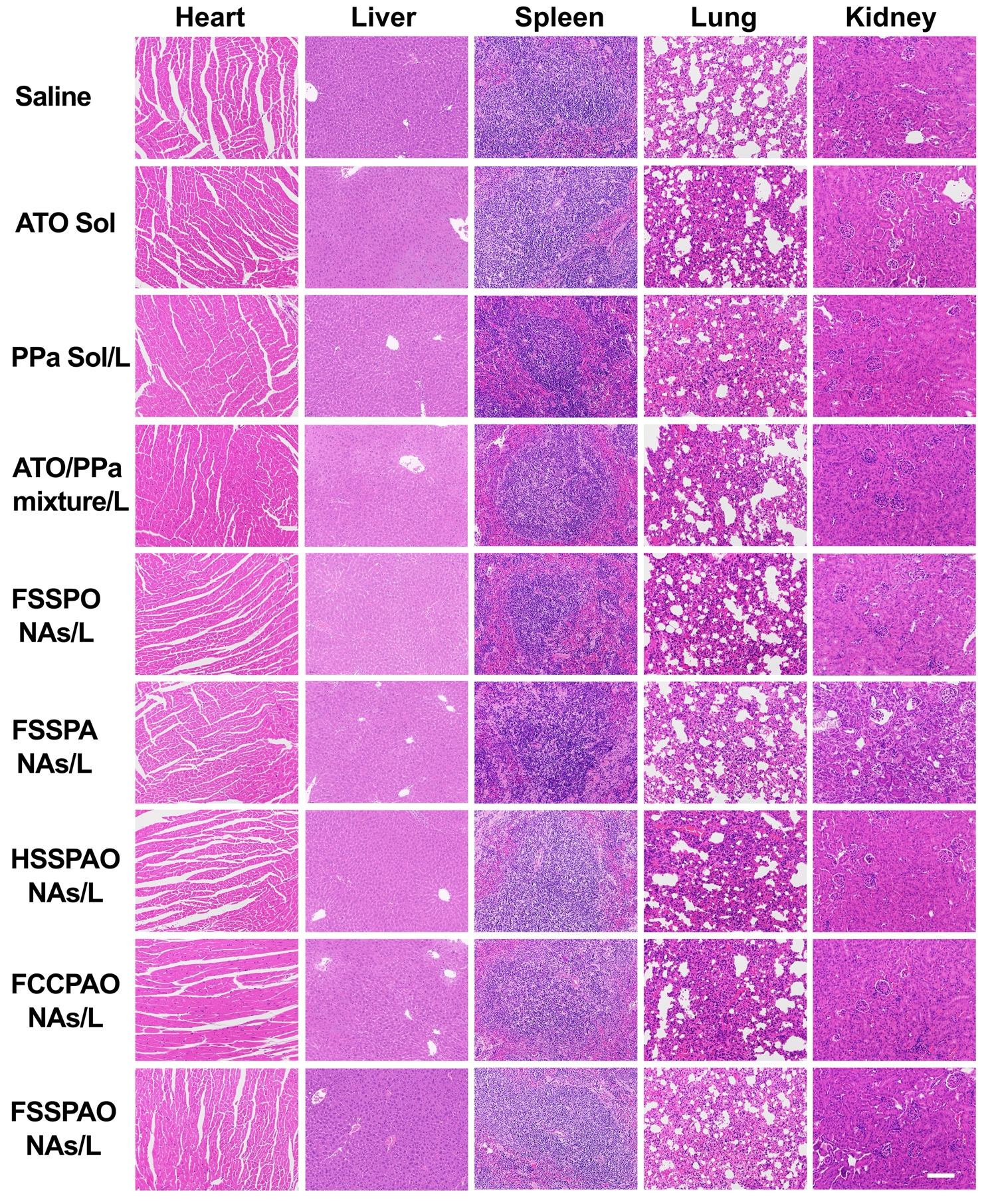


**Figure S25.** H&E staining of the major organs of mice bearing CT26 xenograft colon adenocarcinoma after treatments. Scale bar represents 100 μm.

**Supplementary Tables**

**Table S1.** Particle sizes, PDI and Zeta potentials of PEGylated nanoassemblies.

| **Nanoassemblies** | **Size (nm)** | **PDI** | **Zeta potentials (mV)** |
| --- | --- | --- | --- |
| FSSP NAs | 73.46 ± 1.81 | 0.18 ± 0.07 | -24.6 ± 1.72 |
| FCCP NAs | 70.72 ± 0.71 | 0.18 ± 0.02 | -23.2 ± 0.68 |
| HSSP NAs | 80.64 ± 4.99 | 0.27 ± 0.04 | -25.5 ± 4.65 |

**Table S2.** Particle sizes, PDI and Zeta potentials of PEGylated FSSP/ATO NAs.

| **Nanoassemblies** | **Size (nm)** | **PDI** | **Zeta potentials (mV)** |
| --- | --- | --- | --- |
| 10% | 113.7 ± 4.4 | 0.36 ± 0.03 | -15.2 ± 1.4 |
| 20% | 93.96 ± 1.82 | 0.11 ± 0.02 | -24.6 ± 1.7 |
| 30% | 92.14 ± 0.85 | 0.11 ± 0.05 | -29.2 ± 1.9 |

**Table S3.** Particle sizes and PDI of PEGylated FSSPA NAs at various molar ratios (n=3).

| **FSSP/ATO** | **Size (nm)** | **PDI** |
| --- | --- | --- |
| 4:1 | 100.70 ± 1.56 | 0.26 ± 0.02 |
| 3:1 | 95.20 ± 0.60 | 0.16 ± 0.04 |
| 2:1 | 93.96 ± 1.82 | 0.11 ± 0.02 |
| 1:1 | 100.1 ± 11.81 | 0.34 ± 0.19 |
| 1:2 | 207.8 ± 3.16 | 0.10 ± 0.05 |
| 1:3 | — | — |
| 1:4 | — | — |

**Table S4.** Characterization of nanoassemblies (n=3).

| **Nanoassemblies** | **Size (nm)** | **PDI** | ***Zeta* potentials (mV)** |
| --- | --- | --- | --- |
| FSSPAO NAs | 89.16 ± 6.297 | 0.14 ± 0.03 | -25.3 ± 1.4 |
| FSSPA NAs | 93.96 ± 1.82 | 0.11 ± 0.02 | -24.6 ± 1.7 |
| FCCPA NAs | 87.78 ± 3.06 | 0.13 ± 0.01 | -23.6 ± 3.1 |
| HSSPA NAs | 107.5 ± 0.721 | 0.09 ± 0.02 | -25.8 ± 1.2 |

**Table S5.** Percentages of typical elements in FSSPA NAs.

| **Elements** | **wt%** |
| --- | --- |
| C | 68.36 |
| F | 20.72 |
| Cl | 3.86 |
| S | 4.84 |

**Table S6.** Pharmacokinetic parameters of PPa Sol, FSSPO NAs, FSSPA NAs, HSSPAO NAs, FCCPAO NAs and FSSPAO NAs (n = 5).

| **Formulations** | **^a)^AUC_0-12 h_** | **^b)^C_0.5 h_** | **^c)^t_1/2_** |
| --- | --- | --- | --- |
| PPa Sol | 6.201 ± 0.803 | 2.046 ± 0.193 | 2.568 ± 0.616 |
| FSSPO NAs | 57.037 ± 5.753 | 18.571 ± 1.951 | 3.351 ± 0.594 |
| FSSPA NAs | 60.895 ± 5.994 | 18.234 ± 1.655 | 3.380 ± 0.773 |
| HSSPAO NAs | 13.007 ± 1.324 | 5.727 ± 0.186 | 3.056 ± 0.29 |
| FCCPAO NAs | 76.804 ± 11.068 | 23.244 ± 2.471 | 2.94 ± 0.749 |
| FSSPAO NAs | 56.801 ± 11.304 | 16.834 ± 2.160 | 3.080 ± 0.490 |

**^a)^**Area under the plasma concentration-time curve (μmol/L*h). **^b)^** The plasma concentration at 0.5 h time point (μmol L^-1^). **^c)^**Half-life (h).
